# Using intervarietal substitution lines for the identification of wheat chromosomes involved in early responses to water-deficit stress

**DOI:** 10.1371/journal.pone.0221849

**Published:** 2019-08-29

**Authors:** Karolina Dudziak, Piotr Bulak, Magdalena Zapalska, Andreas Börner, Hubert Szczerba, Justyna Leśniowska-Nowak, Michał Nowak

**Affiliations:** 1 Institute of Plant Genetics, Breeding and Biotechnology, University of Life Sciences in Lublin, Lublin, Poland; 2 Medical University of Lublin, Chair and Department of Biochemistry and Molecular Biology, Lublin, Poland; 3 Department of Natural Environment Biogeochemistry, Institute of Agrophysics, Polish Academy of Sciences, Lublin, Poland; 4 Leibniz Institute of Plant Genetics and Crop Plant Research (IPK), Gatersleben, Germany; 5 Department of Biotechnology, Microbiology and Human Nutrition, Faculty of Food Science and Biotechnology, University of Life Sciences in Lublin, Lublin, Poland; Institute of Genetics and Developmental Biology Chinese Academy of Sciences, CHINA

## Abstract

Water deficit induces reactive oxygen species (ROS) overproduction, which in turn inhibits plant growth and development. High concentrations of ROS disrupt the osmotic balance in plant cells and alter membrane integrity. Chromosomes carrying structural or regulatory genes must be detected to better understand plant response mechanisms to stress. The aim of our study was to identify *Triticum aestivum* L. chromosomes involved in early responses to short-term water-deficit stress (1, 3 and 6 h). In the present study, intervarietal substitution lines of drought-tolerant 'Saratovskaya 29' and sensitive 'Janetzkis Probat' wheat cultivars were examined. We studied the biochemical plant response system and conducted an analysis of catalase, ascorbate peroxidase and guaiacol peroxidase activities, levels of lipid peroxidation and changes in relative water content. Our results determined that the first reaction was a significant increase in guaiacol peroxidase (GPX) activity. However, the strongest impact on plant responses was found for catalase (CAT), which caused a significant decrease in lipid peroxidation (LPO) levels. Our findings indicate that chromosomes 5A, 4B, 6B and 7D are associated with early responses to short-term osmotic stress in wheat.

## Introduction

Cereals are the primary food source for the growing world population, especially in developing countries [1]. Common wheat (*Triticum aestivum* L.) is widely cultivated in every geographical region in the world next to rice and maize [2]. The world’s wheat cultivation area covers 220 million hectares, annually providing approximately 680 million tons of wheat grain [3,4]. From 1980 to 2008, changing climates contributed to global maize and wheat losses of 3.8% and 5.5%, respectively [5]. Drought stress is one of the main limiting factors causing disorders in plant growth and development [6,7]. An increase in mean temperatures and reduced rainfall are the most important agricultural issues affecting many areas of the globe. As a result, physiological, metabolic and morphological plant characteristics change, leading to a decline in crop quality and quantity [8]. The duration and intensity of drought events have a significant impact on reductions of wheat yields, which vary from 10 to 90% of potential levels reached under optimum conditions. Moreover, additional biotic and abiotic stress factors are generally present [9].

Drought leads to oxidative stress in plant cells and induces ROS formation. The peroxidation of cell membranes is the first symptom of oxidative damage caused by stress [10]. Water shortages also affect physiological traits, resulting in relative water content (RWC) changes [11,12,13]. However, plants have developed defense systems to protect cells from toxic ROS. An increase in ROS levels induces biochemical responses by activating or modulating the antioxidant system through several enzymes. The generated H_2_O_2_ is detoxified by peroxidases (APX and GPX) and catalases (CAT). CAT eliminates H_2_O_2_ by converting it into water and molecular oxygen; APX is the first enzyme of the ascorbate-glutathione cycle responsible for H_2_O_2_ detoxification whereas GPX decomposes H_2_O_2_ by oxidizing antioxidants [11,14,15]. Many studies were devoted to describing the characteristics of the antioxidant systems activity under drought treatment in various plant species [13,16,17,18,19]. Studies carried out on plants species of high economic importance such as cereals have frequently been published [10,20]. The alteration of the activities of APX, GPX, and CAT have been observed for *Triticum aestivum* under different environmental conditions (e.g., under cold temperatures [21], in the presence of zinc ions [22] and under drought conditions [13,23]). Many works report chromosomes associated with drought responses [24,25,26,27]. Most previous studies have been based on QTL analyses [28,29,30]. It has been shown that an increase in ABA concentrations can spur a more intense drought response and genes involved in ABA accumulation was identified on chromosome 5A [31]. Genes encoding dehydrins have been found on chromosome 6D in *Aegilops tauschii* [32] and on chromosomes 4A, 5A and 6A in *Triticum monococcum* [33]. Genes associated with drought responses was identified on chromosome 2A of the wheat genome [34]. Moreover, the chromosomes of A and D genomes are the most heavily involved in drought resistance [27]. No significant QTLs have been mapped on chromosomes 2B, 3A, 4A, 4B, 7A and 7B. However, there is still only limited data on how chromosomal regions of the wheat control early responses to short-term water-deficit stress.

The aim of our study was to examine biochemical changes, including antioxidant enzyme activities, relative water content (RWC) levels and membrane damage reflected by lipid peroxidation under water-deficit conditions. Moreover, from intervarietal single chromosome substitution lines (ISCSLs), we identified chromosomes associated with early responses to short-term osmotic stress in wheat.

## Materials and methods

### Plant material and water-deficit stress induction

Intervarietal single chromosome substitution lines (ISCSLs) of wheat (*Triticum aestivum* L.) were examined in this study (S1 Table). 'Saratovskaya 29' (S29), a drought-tolerant cultivar, was used as a recipient, and 'Janetzkis Probat' (JP), a drought-sensitive cultivar, was used as a donor. A set of ISCSL grains was provided by the Leibniz Institute of Plant Genetics and Crop Plant Research (IPK, Gatersleben, Germany).

Grains were disinfected for 4 hours in chlorine gas. To induce germination, grains were incubated for 48 hours at 4°C and then placed on Petri dishes containing filter paper soaked with distilled water for 2 days in the dark at 24°C. Germinating seedlings were transferred to plastic pots containing MS medium. Plants were then grown in hydroponic culture in a growth chamber for 5 days under control conditions (light/dark regime of 16/8 h at 25±3°C, relative humidity of 50±10%, and light intensity during the daytime of 350 μmol m^-2^ s^-1^). Five-day-old seedlings were subjected to osmotic stress by applying 10% of PEG-6000 to MS solution. Wheat seedlings were sampled after 1, 3 and 6 hours of stress treatment and then immediately frozen and stored at -80°C for further analysis. Plants grown in MS medium without PEG were used as a control.

### Homogenate preparation

Homogenates were prepared using the method developed by Balakhnina et al. [35]. Wheat seedling leaves (0.2 g) were homogenized using liquid nitrogen in ice-cold 50 mM sodium phosphate buffer (pH 7.8) containing 5 mM EDTA and 1% (w/v) soluble polyvinylpyrrolidone (PVP). The homogenates were filtered through a nylon cloth. Part of the filtrate was used for a lipid peroxidation analysis and the rest was centrifuged at 11,000 g for 20 min at 4°C (Thermo Fisher Sorvall ST-16). The supernatants were used for the enzymatic assay. The protein content of the samples was estimated using bovine serum albumin (BSA) as a standard [36].

### Lipid peroxidation

Measurements of thiobarbituric acid reactive substance (TBARS) content were collected using Uchiyama and Mihara’s method [37]. The homogenate (0.3 cm^3^) was added to a mixture containing 3 cm^3^ of 1% phosphoric acid, 1 cm^3^ of 0.6% aqueous solution of thiobarbituric acid and 0.1 cm^3^ of aqueous FeSO_4_× 7 H_2_O (2.8 mg cm^3–1^). The samples were incubated at 100°C for 1 h and then quickly cooled on ice. Then, 4 cm^3^ of butanol-1 was added to each sample. After centrifugation at 3,000 g for 10 min, TBARS absorbance was measured at 532 and 600 nm (Shimadzu UV–VIS 160A, Kyoto, Japan). TBARS concentrations were calculated using a coefficient of extinction of 156 × mM^-1^ cm^-1^ and were expressed as μmol/g fresh weight (FW).

### Determination of antioxidant enzyme activity

CAT activity was measured by estimating H_2_O_2_ degradation, which was identified as a decrease in absorbance at 240 nm (extinction coefficient of 39.4 mM^−1^ cm^−1^) [38]. The reaction mixture contained 0.036% H_2_O_2_ solution in sodium phosphate buffer (pH 7.8) and 50 mm^3^ of plant extract in a final volume of 3 cm^3^. Enzyme activity was expressed as μmol H_2_O_2_ mg protein^-1^ min^-1^.

Measurements of APX (EC 1.11.1.11) activity were performed following Nakano and Asada’s method [39]. The reaction mixture (3 cm^3^) consisted of 50 mM phosphate buffer (pH 7.5), 0.5 mM ascorbate, 0.1 mM EDTA and 0.1 cm^3^ of enzyme extract. The reaction was initiated by adding 0.1 cm^3^ of 0.1 mM. H_2_O_2._ The decrease in absorbance as a result of ascorbate oxidation was measured at 290 nm (extinction coefficient of 2.8 mM cm^-1^). APX activity was expressed as μmol ascorbate oxidized mg min^-1^ protein^-1^.

GPX activity (EC 1.11.1.9) was assayed based on the modified method described by Balakhnina et al. [35]. The reaction mixture (3 cm^3^) contained 50 mM phosphate buffer (pH 7.5), 0.1 cm^3^ of 0.15% H_2_O_2_ and 0.1 cm^3^ of plant extract. The reaction was initiated by adding 0.1 cm^3^ of 56 mM guaiacol. The increase in absorbance as a result of guaiacol conversion into its oxidized form (tetraguaiacol) was measured at 470 nm. GPX activity was determined using the extinction coefficient for tetraguaiacol (26.6 mM^-1^ cm^-1^).

### Relative water content (RWC)

Relative water content (RWC) was measured in control and stressed seedlings as described earlier [11,40,41]. Fully expanded leaves were immediately cut and weighed as fresh weight (FW). The seedlings were then soaked for 4 h in distilled water in the dark, and the turgid weight was measured. The samples were subsequently dried for 24 h at 80°C in a hot-air oven and the total dry weight (DW) was recorded. RWC was calculated according to the following formula: RWC (%) = ((FW-DW)/(TW-DW))*100 [42].

### Statistical analysis

The experiments were repeated in two full independent biological replications and each data point was measured as the mean of three technical replications (n = 6). A one-way analysis of variance (ANOVA) and Tukey’s posttest were used to compare the means of controls and stress-treated plants across the same substitution lines. Dunnett's test was applied to estimate significant differences between the tested lines and S29; differences measured at p < 0.05 were considered significant. Correlations of biochemical changes were estimated using Pearson’s correlation coefficient. In order to determine the main sources of variability, principal component analysis (PCA) method was applied. All data were analyzed using Statistica 13.1 (Dell Software).

## Results

### Lipid peroxidation

**Fig 1:**
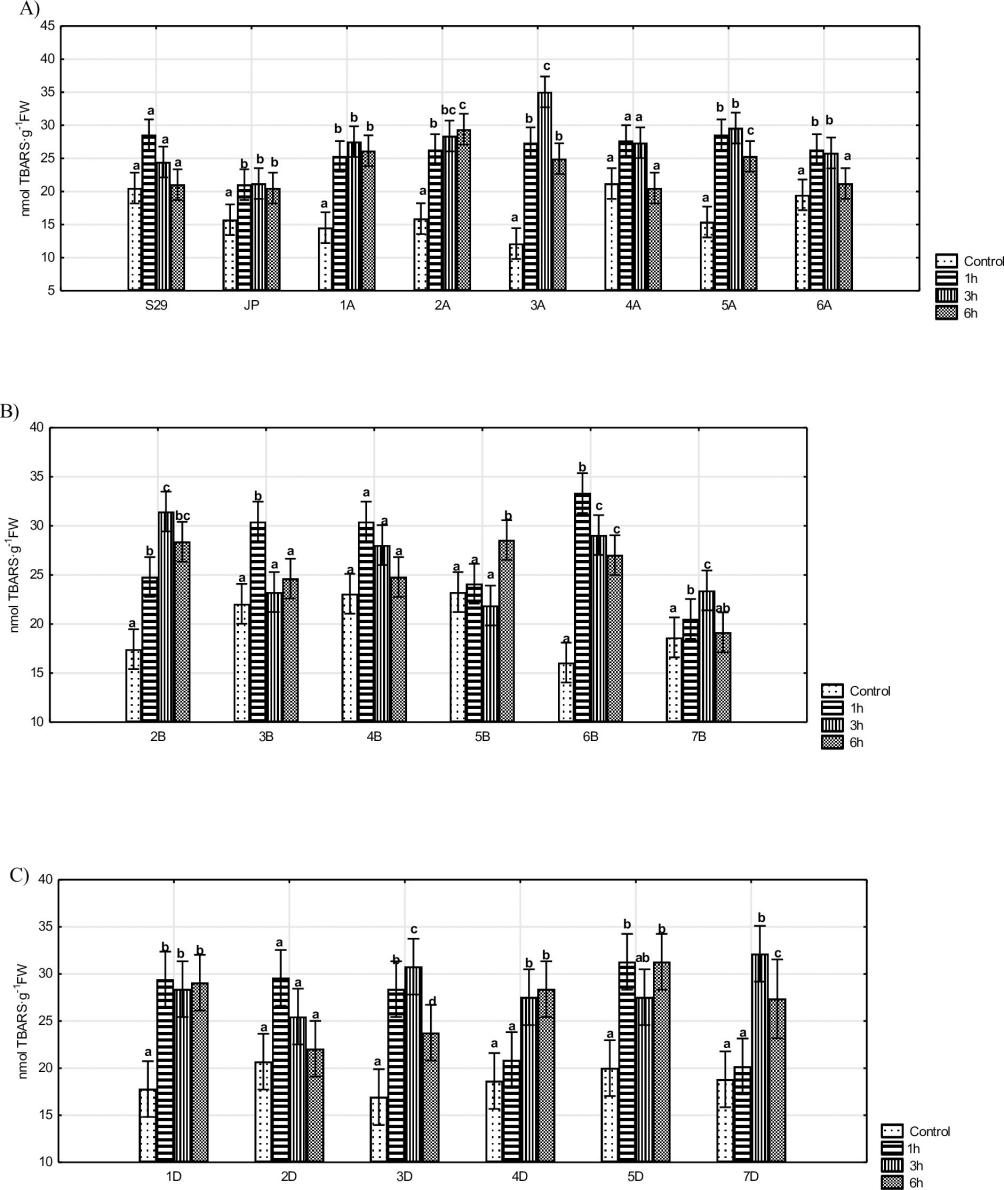
Levels of lipid peroxidation expressed in terms of TBARS concentration in S29(JP) substitution lines during 1, 3 and 6 h of 10% PEG treatment and in non-exposed plants. A) S29; JP; lines with substitution of A genome chromosomes; B) lines with substitution of B genome chromosomes; C) lines with substitution of D genome chromosomes. Bars represent confidence interval (CI). Letters (a, b, c) indicate significant differences at p<0.05 according to Tukey’s multiple range test, means with the same letter are not significantly different.

Water stress induced lipid peroxidation was observed in most of the tested lines and was expressed by higher levels of TBARS content (Fig 1). Only S29 and lines of homoeologous group 4 (4A, 4B) and line 2D did not show any significant changes in lipid peroxidation levels over 6 h of stress. However, the main trend observed for most of the remaining lines (lines containing chromosomes belonging to homoeologous groups 3, 6 and 7) involved an immediate response and an increase in LPO levels within the first hours of PEG treatment. TBARS levels subsequently decreased. Drought-sensitive cultivar JP and lines with the substituted homoeologous group 1 chromosomes showed an increase in LPO after 1 h, which then remained at stable levels. The strongest changes in LPO levels, which continuously increased over 6 h of the experiment, were observed for line 4D. The most stable level of lipid peroxidation was observed for lines with substituted A genome chromosomes. Significant differences between the drought-tolerant (S29) and drought-sensitive (JP) cultivars were observed only after 1 h of stress. The substitution of chromosomes 6B and 7D had a stronger impact on LPO levels than the recipient (S1, S2 and S3 Figs).

### Antioxidant enzyme activity

**Fig 2:**
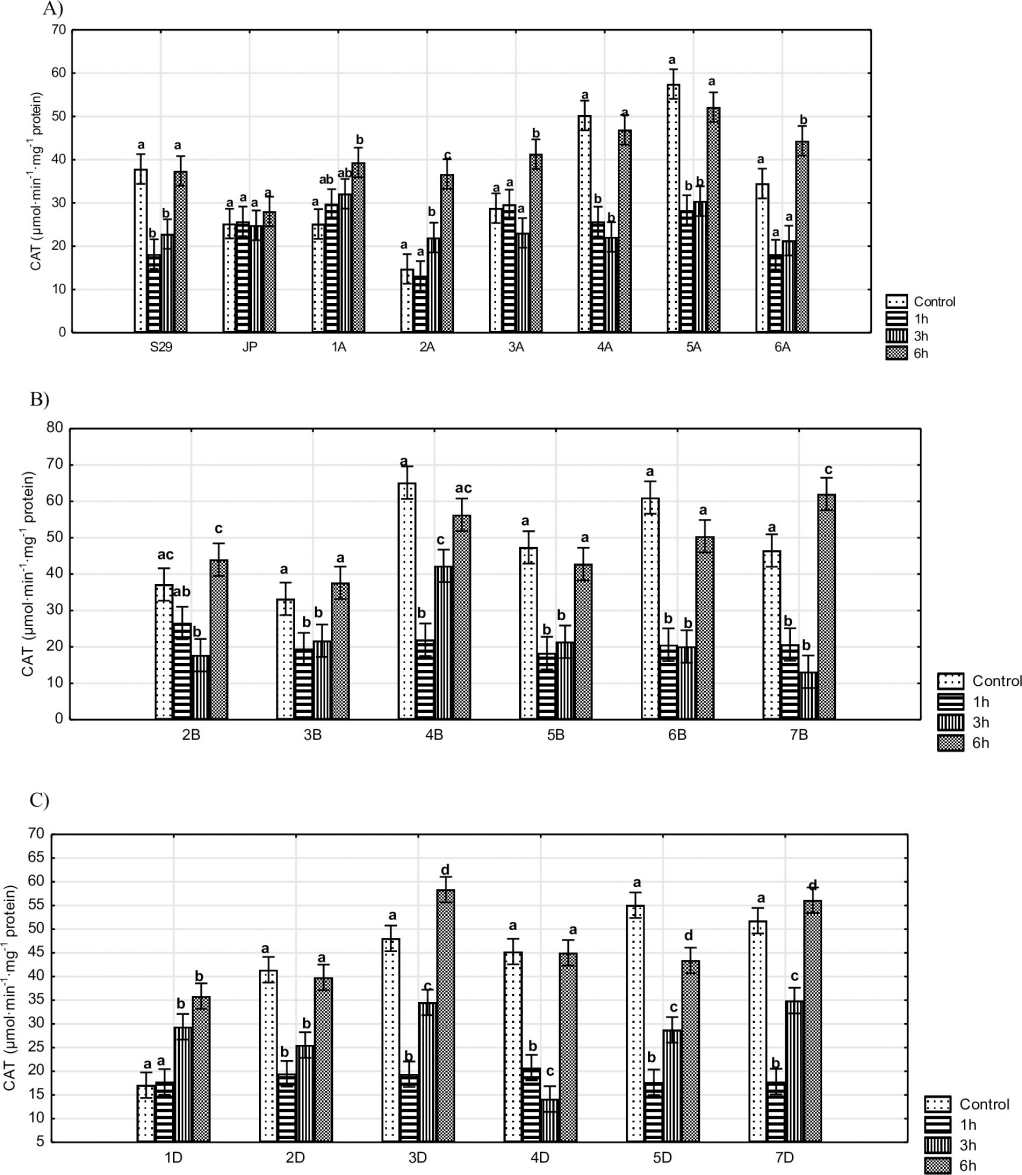
The activity of CAT in S29(JP) substitution lines during 1, 3 and 6 h of 10% PEG treatment and in non-exposed plants. A) S29; JP; lines with substitution of A genome chromosomes; B) lines with substitution of B genome chromosomes; C) lines with substitution of D genome chromosomes. Bars represent confidence interval (CI). Letters (a, b, c) indicate significant differences at p<0.05 according to Tukey’s multiple range test, means with the same letter are not significantly different.

The same characteristic patterns of CAT activity were observed in the majority of lines (Fig 2). The initial level of catalase decreased significantly over the first hours of stress and then increased after 6 h. This trend was recorded for all lines (except for 1A, 1D, 2A, and 3A) and for drought-tolerant cultivar S29. The remaining lines did not show significant changes after 1 h of stress, but CAT activity levels increased after 3 h (1D and 2A) and 6 h (for 1A, 1D, 2A, and 3A) of stress. No changes were observed for JP over 6 h of osmotic stress. Lines 1A, 4A, 5A, 4B and 7D showed the strongest differences in CAT activity relative to the recipient (S29) (S4, S5, S6 and S7 Figs). Significant differences between the drought-tolerant and sensitive cultivars were observed after 1 and 6 h of water deficit and under optimum conditions.

**Fig 3:**
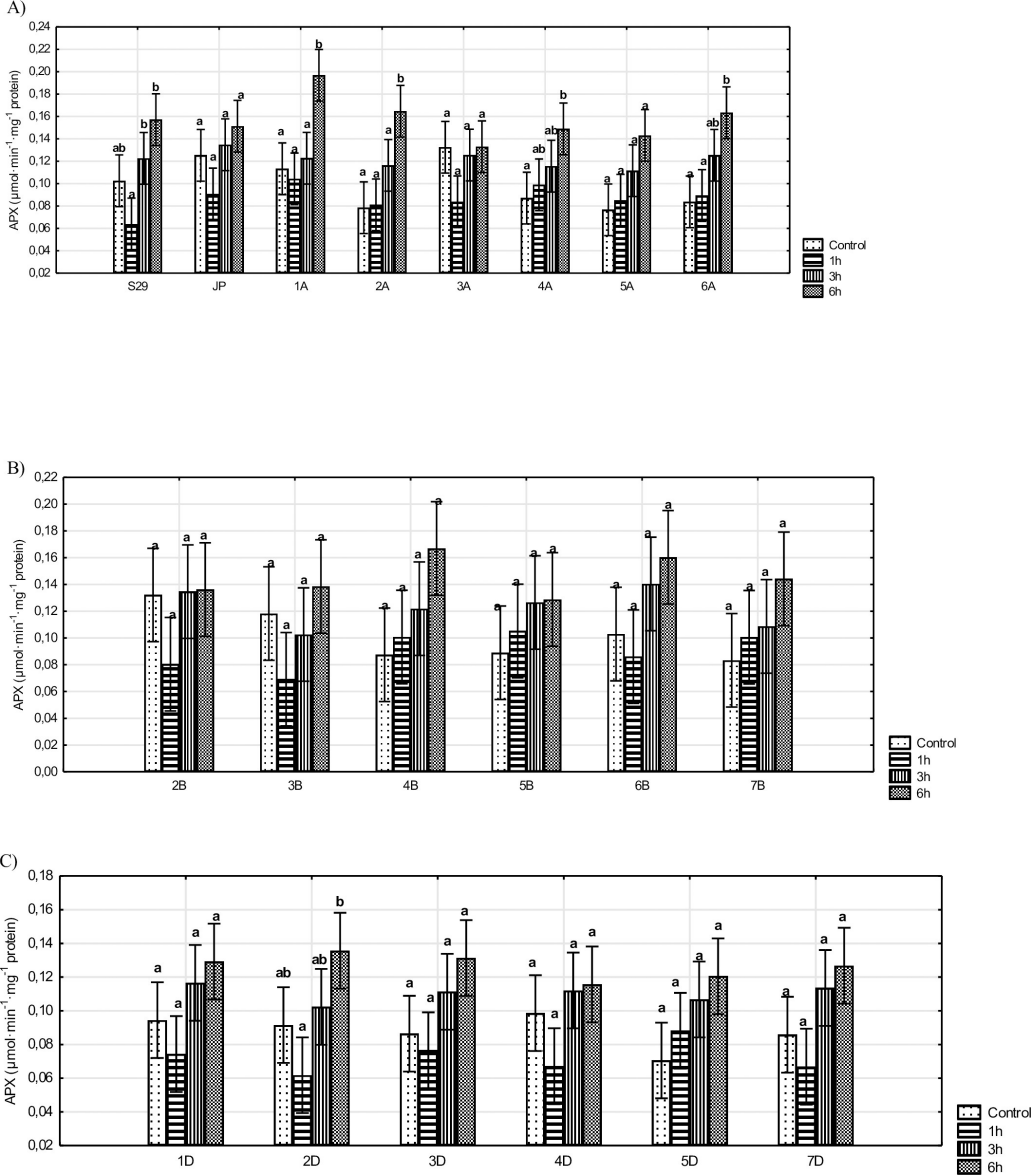
The activity of APX in S29(JP) substitution lines during 1, 3 and 6 h of 10% PEG treatment and in non-exposed plants. A) S29; JP; lines with substitution of A genome chromosomes; B) lines with substitution of B genome chromosomes; C) lines with substitution of D genome chromosomes. Bars represent confidence interval (CI). Letters (a, b, c) indicate significant differences at p<0.05 according to Tukey’s multiple range test, means with the same letter are not significantly different.

The results of the APX activity analysis indicate that significant changes were only observed for lines with substituted A genome chromosomes (except for 3A and 5A), 2D and S29 (Fig 3). However, an increase in APX activity levels was observed after only 6 h of stress. Other lines did not show any changes in the activity of this enzyme. Furthermore, all tested lines and JP did not show any significant differences relative to the recipient (S29) (S4, S8, S9 and S10 Figs).

**Fig 4:**
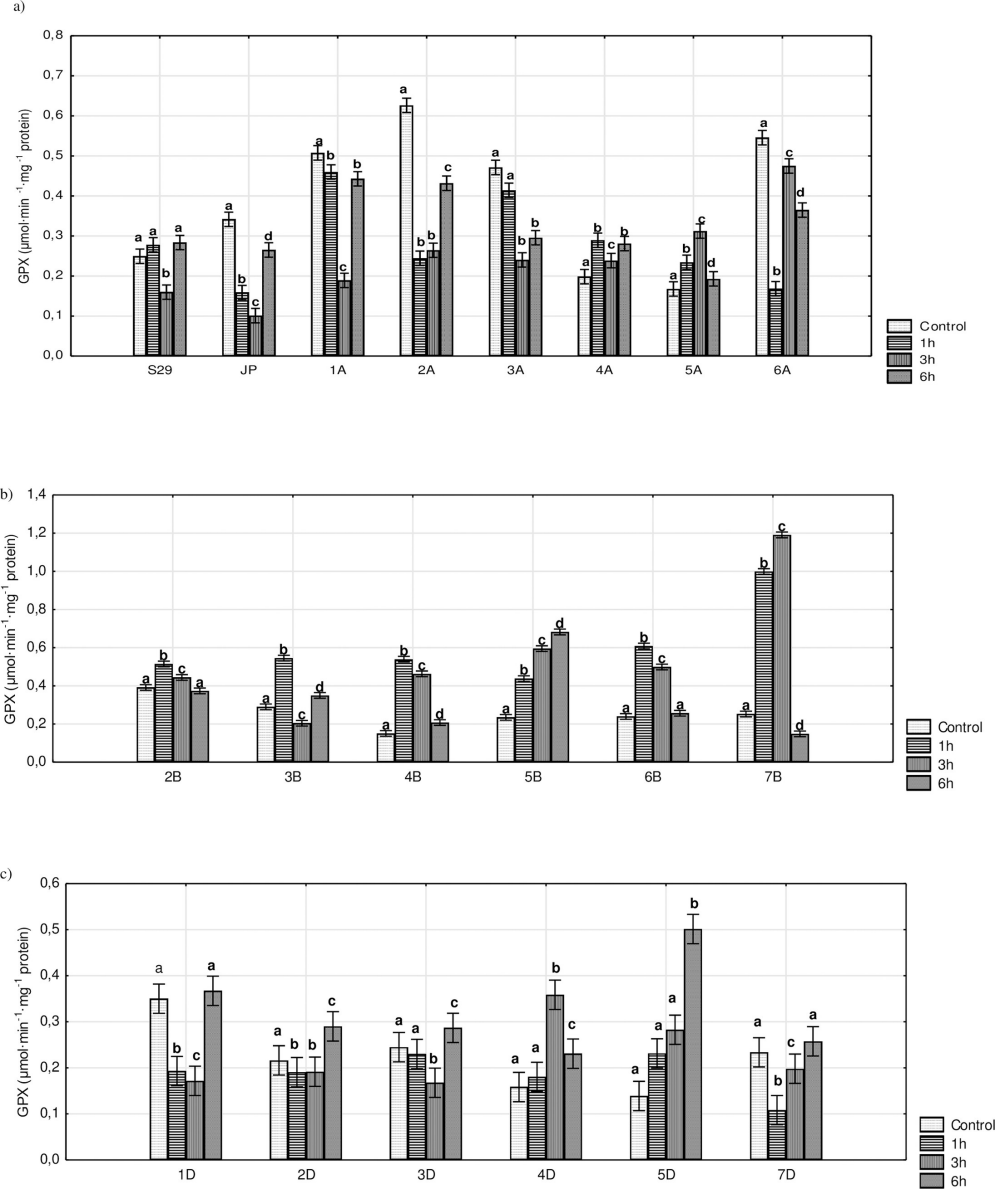
The activity of GPX in S29(JP) substitution lines during 1, 3 and 6 h of 10% PEG treatment and in non-exposed plants. A) S29; JP; lines with substitution of A genome chromosomes; B) lines with substitution of B genome chromosomes; C) lines with substitution of D genome chromosomes. Bars represent confidence interval (CI). Letters (a, b, c) indicate significant differences at p<0.05 according to Tukey’s multiple range test, means with the same letter are not significantly different.

The analysis of GPX activity did not reveal any characteristic trends (Fig 4). For lines with substituted B genome chromosomes (except for 5B) and for 4D and 5A lines, an increase in GPX activity was observed over the first hours of stress, which significantly diminished after 6 h. Lines with substituted D genome chromosomes showed a constant increase in activity (5D) or an initial decrease followed by an increase after 6 h (1D, 2D, 3D, and 7D). The same pattern of activity was observed in S29. A similar trend was observed for lines with substituted A genome chromosomes and JP. However, levels of activity for these forms were still lower after 6 h of stress than for the untreated plants. Significant differences were observed for many of the tested lines (5A, 6A, 2B, 4B, 5B, 6B, 7B, 4D, and 5D) at each time point of PEG treatment relative to S29. The results indicate that lines containing chromosomes of homoeologous groups 5, 6 and 7 had the strongest impacts on GPX activity. Significant differences were found between S29 and JP after 1 h of stress and under control conditions (S4, S11, S12 and S13 Figs).

Furthermore, the performed analysis shows that CAT activity is positively correlated with APX and negatively correlated with GPX and LPO (Table 1).

**Table 1:** The Pearson's correlation between APX, CAT, GPX activity and LPO in S29(JP) substitution lines.

Trait	Correlation p < .05000 N = 160			
	APX	CAT	GPX	LPO
APX	1.000000	0.290520*	0.062702	0.030515
CAT	0.290520*	1.000000	-0.308663*	-0.263656*
GPX	0.062702	-0.308663*	1.000000	0.025849
LPO	0.030515	-0.263656*	0.025849	1.000000

* indicates significant differences compared at p<0.05

### Relative water content

**Fig 5:**
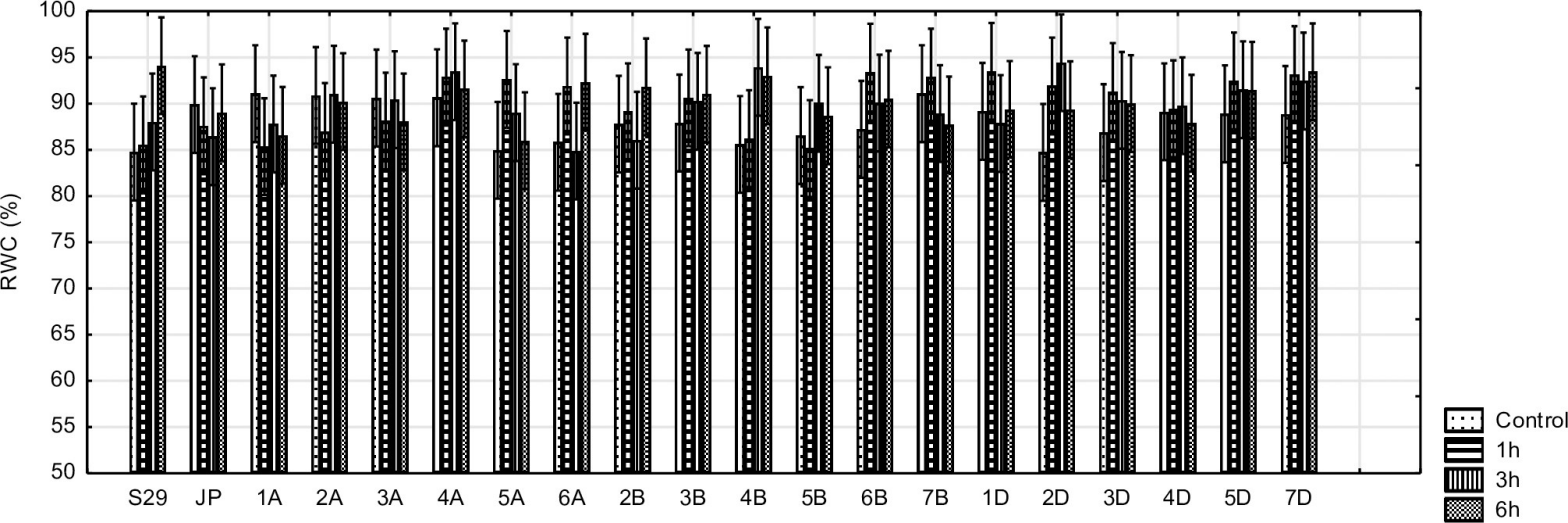
RWC in S29(JP) substitution lines during 1, 3 and 6 h of 10% PEG treatment and in non-exposed plants. Bars represent confidence interval (CI).

The obtained results do not show any significant changes in RWC for the tested lines treated with 10% PEG for 6 h. Water content levels were similar across all lines and varied between 85% and 95%. All changes in RWC were not statistically significant. No differences between the donor (S29) and tested lines were detected (Fig 5).

### Principal component analysis (PCA)

**Fig 6:**
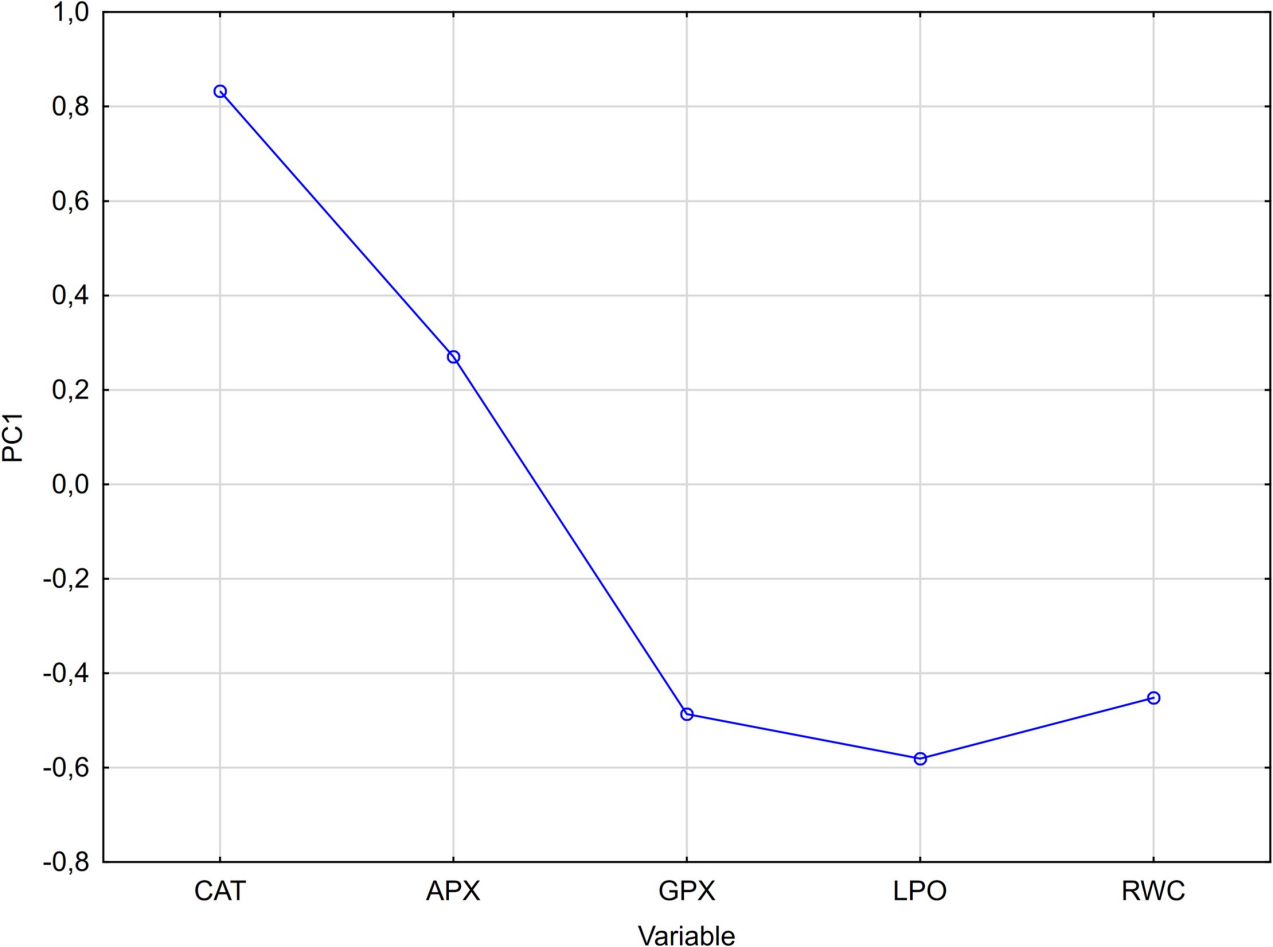
Loading lineplot for PC1.

**Fig 7:**
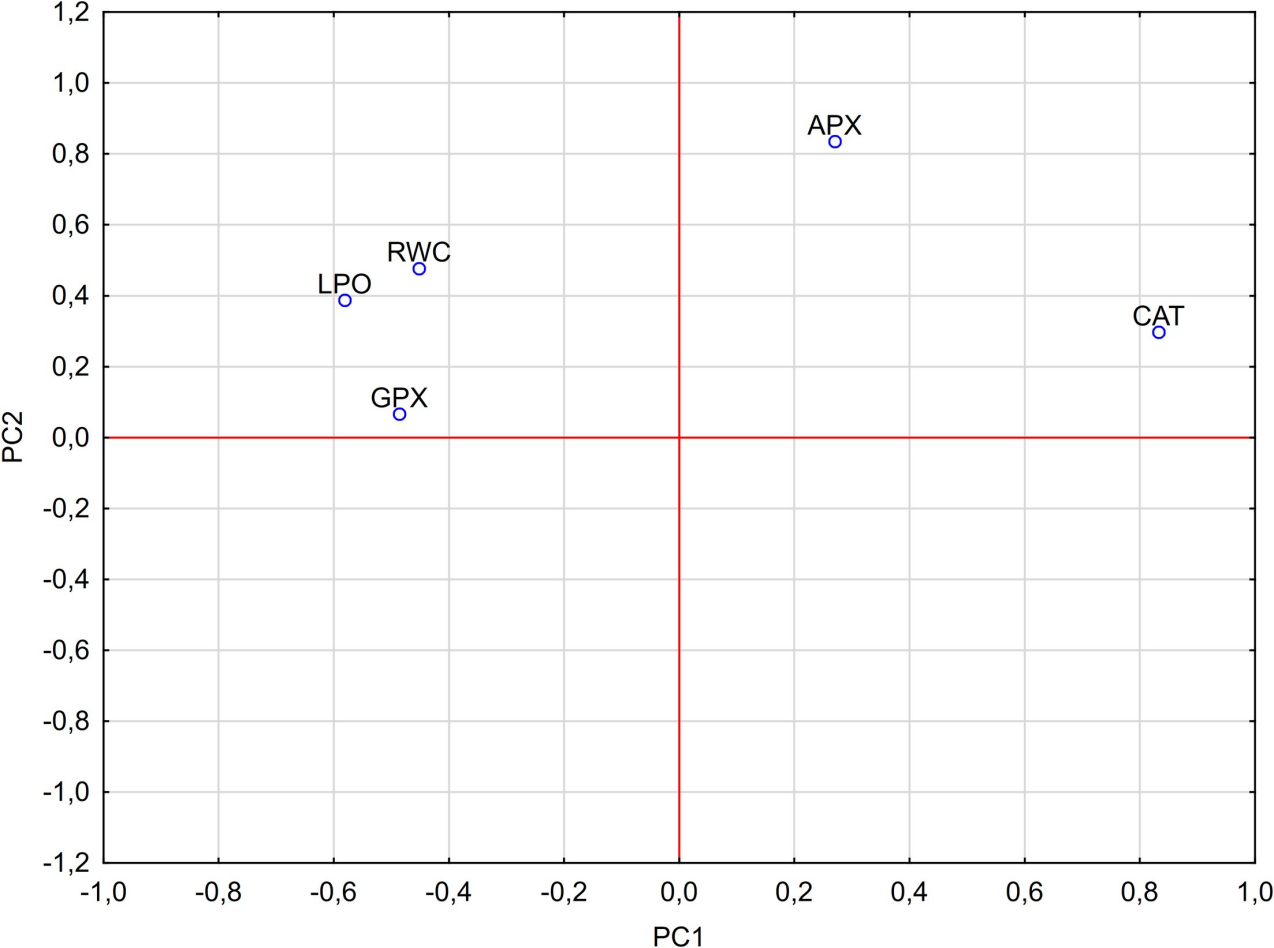
Loading scatterplot PC1 vs. PC2 for analyzed variables.

The PCA analysis revealed that APX activity is the least influential in determining the PC1 while CAT activity plays the most important role (Fig 6). The first four PCs represented 92.96% of the total variance (PC1: 30.89%, PC2: 23.35%, PC3: 20.96%, PC4: 17.76%). Analysis of the loading factors between principal components showed that the activity of CAT and APX are the most influential variables in determining the PCA model (Fig 7).

## Discussion

The formation of ROS is responsible for the stress-induced peroxidation of lipids and for membrane damage. Some compounds react with thiobarbituric acid (TBA), e.g., MDA, and form products called thiobarbituric acid reactive substances (TBARS) [43]. Total quantity of TBARS reflects the level of LPO [11]. In our study, most of the tested lines showed an increase in LPO levels after 1 or/and 3 h and underwent a significant decline after 6 h of water-deficit stress what indicates that the third hour may constitute the key period of responses to water-deficit stress during the seedling stage. Moreover, lipid peroxidation of membranes was not severe at this time. Drought did not have a significant impact on plant conditions and plants were able to acclimate. The results confirm previous data on the application of short-term stress (stress measured in hours) in *Eremosparton songoricum* [44], *Setaria italica* [11], barley [10], *Pisum sativum* L. [45]. Our study demonstrates that unchanged LPO levels observed are associated with plant tolerance to osmotic stress. No changes in lipid peroxidation were observed in *Cleome gynandra* [46] or for a tolerant maize cultivar [47]. Moreover, our results reveal a higher level of LPO in S29 than in JP what can indicate that different adaptive mechanisms are involved in the regulation of its redox status. Furthermore, the drought-tolerant cultivar showed higher levels of membrane integration under osmotic stress, which are used as a direct indicator of dehydration stress tolerance. Similar observations have been reported on drought-sensitive and tolerant wheat cultivars [48] or *Setaria italica* L. [11]. Several studies have been conducted on *Triticum aestivum* responses to long-term drought [13,49]. Previous reports show a continuous rise in LPO levels occurring after 3, 6, 9 and 10 d of drought. However, these data do not describe early plant reaction occurring directly after the introduction of stress. From the presented results and previous studies, we suggest that wheat seedlings can respond to stress induction immediately by activating antioxidant enzymes, but they cannot endure prolonged periods of drought stress.

Our study demonstrates that major patterns of CAT activity undergo a significant decline after 1 and 3 h followed by an increase. Similar observations have been made for *Eremosparton songoricum* under drought conditions [44] and for maize seedlings treated with sorbitol [50]. In the present study the level of CAT activity significantly declines during the first hours of stress due to the osmotic shock caused by H_2_O_2_ accumulation. The antioxidant system has not been yet activated. The same trend has also been observed in the 'Rum' cultivar of barley during long periods of stress [17]. In our study, a significant increase in CAT activity was observed after a longer water deficit period (6 h). Similar reaction has been described for tobacco after 10 h of 10% PEG treatment [51] and for a tolerant maize cultivar after 72 h of drought [47].

A different pattern of CAT activity was observed for substitution lines 4A, 6A, 4B and 7D after long-term periods of water shortage relative to the recipient [26]. Based on our results and previous studies, these chromosomes are associated with wheat responses expressed by changes in CAT activity occurring during short- and long-term periods of water deficit. Moreover, Osipova et al. [26] reported an increase in enzyme activity for lines 1A, 6A and 7B and no significant changes in lines 4A, 5A, 5B, 2B, 6B and 2D, which is consistent with the results of our study. Thus, we assume that prolonged osmotic stress treatment should not change the observed pattern of CAT activity for these lines.

We did not observe any changes in APX activity for most of the tested lines. Our results confirm the results of an earlier study performed on drought-sensitive wheat cultivars subjected to mild (-0.4 MPa) osmotic stress for 8 d [16]. Moreover, no induction of APX activity has been observed for *Pisum sativum* L. [45], maize [50], *Koeleria macrantha* [52], rice [53], *Phaseolus vulgaris* [54], or the 'Rum' cultivar of barley [17] in the first hours of stress. According to Hernándeza and Almansa [45], APXs are present in mitochondria and peroxisome membrane fractions, where they play a major function in H_2_O_2_ scavenging. This scavenging system can prevent increases in H_2_O_2_ levels in the cytosol during periods of optimum growing conditions and under certain plant-stress conditions as H_2_O_2_ concentrations increase. This lack of change in enzyme activity can be attributed to a high level of activity observed in plants under control conditions. Moreover, Mittler and Zilinskas [55] found that minor changes in APX activity observed in response to drought are sufficient for scavenging elevated levels of H_2_O_2_ forming under these stress conditions. However, based on previous reports, we believe that prolonged drought treatment should cause an increase in APX activity, which was observed in wheat after 6 d [49] and in the 'Yarmouk' barley cultivar after 9 d [17]. In our study, we observed an enhanced level of APX activity in drought-tolerant cultivar ‘Saratovskaya 29.’ According to Chugh et al. [47], increased APX activity levels in tolerant forms might constitute an adaptive response to a higher level of ROS. Similar observations have been reported for drought-resistant maize [47] and wheat [16]. Osipova et al. [26] observed a significant increase in APX activity for lines 1A, 2A and 4A during periods of long-term stress. Moreover, these authors did not observe any changes in APX activity for most lines substituting B and D genome chromosomes. This study indicates that prolonged periods of water deficit should not cause any changes in APX activity in these lines.

In our study, we did not find major trends of GPX activity. Variable patterns of GPX activity have also been observed in different genotypes of wheat [56] and maize [47]. From previous data, we suggest that a longer drought treatment period would spur a continuous increase in GPX activity in S29 but no changes in JP [13]. Furthermore, the drought-resistant cultivar exhibited a higher level of GPX activity compared to the sensitive one, which has also been described for wheat by Sheoran et al. [13]. The latter authors suggest that different wheat genotypes have discrete water stress thresholds and therefore use different physiologically adaptive mechanisms to regulate their redox status. In our study, a decline in GPX activity observed in the first hours of stress was recorded for most of the tested lines with the substitution of A and D genome chromosomes. Similar outcomes have been described for *Brassica* after 3 h of heat stress [57]. We found 6 h to be an insufficient amount of time to enhance GPX activity in lines substituting A genome chromosomes. The limited activity of GPX observed in these lines suggests that other enzymes play the main role in the antioxidant system (CAT and APX) during the short-term stress treatment. A significant increase in GPX activity after 1 h of stress was observed for lines with substituting B genome chromosomes and 5A and 4D lines. Rapid stress responses expressed by an immediate increase in GPX activity have also been found for *Vigna aconitifolia* [19] and *Eremosparton songoricum* [44]. According to Chugh et al. [47], peroxidases are often the first enzymes, and their activity levels change under stress. Previous data obtained for maize [47,50] and wheat [49,58,59] suggests that longer periods of stress should increase GPX activity. Li et al. [44] suggested that the period revealing the highest activity in all antioxidant enzymes may constitute the key point of responses to drought stress during the seedling stage. According to Chakraborty and Pradhan [49], CAT and GPX enzyme activities are considered an important anti-drought mechanism in coping with oxidative stress during water deficit periods. Furthermore, Sharma and Dubey [60] suggested that an increase in peroxidase activity in stressed seedlings may be correlated with oxidation reactions associated with an increase of peroxide and free radical content in plant cells.

The results of our analysis reveal a significant correlation between CAT and APX but a negative one between CAT and GPX. A negative correlation between CAT and GPX has also been found for maize [50], wheat [59] and *Brassica* [57]. According to our results, declines in GPX activity observed suggest that CAT and APX perform central functions in the antioxidant mechanism. Furthermore, a positive correlation between CAT and APX was observed in wheat treated with PEG [59] and in *Kentucky bluegrass* [61]. The present study reveals a negative correlation between CAT and LPO consistent with previous reports on *Eremosparton songoricum* [44] and *Kentucky bluegrass* [61].

In the current study, we did not observe any significant changes in RWC over 6 h of osmotic stress. Moreover, no significant differences in RWC were observed among the tested lines. From the obtained results and previous studies, we suggest that 6 h of 10% PEG treatment should not lead to physiological changes in plants attributed to alterations in RWC. Our results are in line with many reports on wheat [12,56] and other plant species such as *Cleome gynandra* [46] and *Phaseolus acutifolius* [54]. According to Bandurska et al. [10], RWC serves as a good indicator of a plant’s ability to avoid dehydration by osmotic adjustment. Most studies have demonstrated that long-term periods of drought cause a decrease in RWC in wheat after 2 days [12], 3 days [49] and 5 days [14]. From previous reports, we suggest that in our experiments, significant changes in RWC could be observed after at least 48 h. Ozfidan et al. [62] proved that the period of stress has a significant impact on RWC. Moreover, plant developmental stage also affects RWC. Declines in RWC occurring during an earlier growth stage are generally less pronounced than those occurring in later growth stages [63]. RWC is also shaped by the concentration of PEG. Variable responses to stress induced by different PEG levels have been observed in wheat [48] and maize [64]. Based on previous studies, the 10% PEG induces mild water-deficit conditions that do not affect RWC and that result in adaptation to drought through effective osmoregulation. The tested lines exhibited a capacity for osmoregulation, which enabled them to maintain a relatively high volume of turgor protoplasts and high levels of activity in the photosynthetic apparatus [48]. In our study, an insignificant increase in RWC values was observed for certain lines. An increase in RWC has also been observed in *Arabidopsis thaliana* [62] and tomato plants [65] under drought. These authors suggest that such an increase in RWC may be related to the accumulation of a larger volume of proline.

In the present study, we identified chromosomes associated with early wheat responses to short-term stress from a set of S29 substitution lines (JP) showing differing degrees of tolerance to water deficit. We found that the substitution of chromosomes 6B and 7D spurred the most significant changes in LPO to the recipient. It indicates that chromosomes 1A, 4A, 5A, 4B and 7D include genes involved in CAT activity. Genes involved in GPX activity are associated with chromosomes belonging to homologous groups 5 (5A, 5B, and 5D) and 4 (4B and 4D) and to chromosomes 6A, 2B and 7B.

## Conclusions

Our study reveals a complex system of plant responses to short-term water-deficit stress. The first reaction of the tested wheat lines included an increase in GPX activity. However, catalase had the strongest impact on plant responses, as it caused a significant decline in LPO levels. In the current study, we found that chromosomes 5A, 4B, 6B and 7D are associated with early wheat responses to short-term stress. The substitution of these chromosomes spurred the most significant changes in the activity of the tested enzymes and in lipid peroxidation relative to S29. Therefore, structural and regulatory genes involved in rapid plant reactions may be positioned on these chromosomes.

## Supporting information

S1 Fig **Changes of lipid peroxidation expressed in terms of TBARS concentration in lines with substitution of A genome chromosomes (a-f) compared to S29 during 1, 3 and 6 h of 10% PEG treatment and in non-exposed plants. Bars represent 95% confidence intervals (CI).** *indicates significant differences compared to S29 at p<0.05 according to Dunnett's test.
(TIF)

S2 Fig **Changes of lipid peroxidation expressed in terms of TBARS concentration in lines with substitution of B genome chromosomes (a-f) compared to S29 during 1, 3 and 6 h of 10% PEG treatment and in non-exposed plants.** Bars represent confidence interval (CI). *indicates significant differences compared to S29 at p<0.05 according to Dunnett's test.
(TIF)

S3 Fig **Changes of lipid peroxidation expressed in terms of TBARS concentration in lines with substitution of D genome chromosomes (a-f) compared to S29 during 1, 3 and 6 h of 10% PEG treatment and in non-exposed plants.** Bars represent confidence interval (CI). *indicates significant differences compared to S29 at p<0.05 according to Dunnett's test.
(TIF)

S4 Fig **Changes of a) lipid peroxidation expressed in terms of TBARS concentration b) CAT activity c) APX activity d) GPX activity in JP compared to S29 during 1, 3 and 6 h of 10% PEG treatment and in non-exposed plants.** Bars represent confidence interval (CI). *indicates significant differences compared to S29 at p<0.05 according to Dunnett's test.
(TIF)

S5 Fig **Changes of CAT activity in lines with substitution of A genome chromosomes (a-f) compared to S29 during 1, 3 and 6 h of 10% PEG treatment and in non-exposed plants.** Bars represent 95% confidence intervals (CI). *indicates significant differences compared to S29 at p<0.05 according to Dunnett's test.
(TIF)

S6 Fig **Changes of CAT activity in lines with substitution of B genome chromosomes (a-f) compared to S29 during 1, 3 and 6 h of 10% PEG treatment and in non-exposed plants.** Bars represent confidence interval (CI). *indicates significant differences compared to S29 at p<0.05 according to Dunnett's test.
(TIF)

S7 Fig **Changes of CAT activity in lines with substitution of D genome chromosomes (a-f) compared to S29 during 1, 3 and 6 h of 10% PEG treatment and in non-exposed plants.** Bars represent confidence interval (CI). *indicates significant differences compared to S29 at p<0.05 according to Dunnett's test.
(TIF)

S8 Fig **Changes of APX activity in lines with substitution of A genome chromosomes (a-f) compared to S29 during 1, 3 and 6 h of 10% PEG treatment and in non-exposed plants.** Bars represent 95% confidence intervals (CI). *indicates significant differences compared to S29 at p<0.05 according to Dunnett's test.
(TIF)

S9 Fig **Changes of APX activity in lines with substitution of B genome chromosomes (a-f) compared to S29 during 1, 3 and 6 h of 10% PEG treatment and in non-exposed plants.** Bars represent confidence interval (CI). *indicates significant differences compared to S29 at p<0.05 according to Dunnett's test.
(TIF)

S10 Fig **Changes of APX activity in lines with substitution of D genome chromosomes (a-f) compared to S29 during 1, 3 and 6 h of 10% PEG treatment and in non-exposed plants.** Bars represent confidence interval (CI). *indicates significant differences compared to S29 at p<0.05 according to Dunnett's test.
(TIF)

S11 Fig **Changes of GPX activity in lines with substitution of A genome chromosomes (a-f) compared to S29 during 1, 3 and 6 h of 10% PEG treatment and in non-exposed plants.** Bars represent 95% confidence intervals (CI). *indicates significant differences compared to S29 at p<0.05 according to Dunnett's test.
(TIF)

S12 Fig **Changes of GPX activity in lines with substitution of B genome chromosomes (a-f) compared to S29 during 1, 3 and 6 h of 10% PEG treatment and in non-exposed plants.** Bars represent confidence interval (CI). *indicates significant differences compared to S29 at p<0.05 according to Dunnett's test.
(TIF)

S13 Fig **Changes of GPX activity in lines with substitution of D genome chromosomes (a-f) compared to S29 during 1, 3 and 6 h of 10% PEG treatment and in non-exposed plants.** Bars represent confidence interval (CI). *indicates significant differences compared to S29 at p<0.05 according to Dunnett's test.
(TIF)

S1 Table List of the intervarietal single chromosome substitution lines (ISCSLs) used in the study. (PDF)

## Abbreviations

ABA: Abscisic Acid
APX: Ascorbate Peroxidase
BSA: Bovine Serum Albumin
CAT: Catalase
EDTA: Ethylenediaminetetraacetic Acid
FAO: Food and Agriculture Organization
GPX: Guaiacol Peroxidase
ISCSLs: Inter-varietal Single Chromosome Substitution Lines
LPO: Lipid Peroxidation
MDA: Malondialdehyde
PEG: Polyethylene glycol
PVP: Polyvinylpyrrolidone
QTL: Quantitative Trait Loc
ROS: Reactive Oxygen Species
RWC: Relative Water Content
TBARS: Thiobarbituric Acid Reactive Substances
