# Supplementary figures and images for: Using intervarietal substitution lines for the identification of wheat chromosomes involved in early responses to water-deficit stress
Source: PLoS One. 2019 Aug 29;14(8):e0221849. doi: 10.1371/journal.pone.0221849 (PMC6715202; doi:10.1371/journal.pone.0221849)

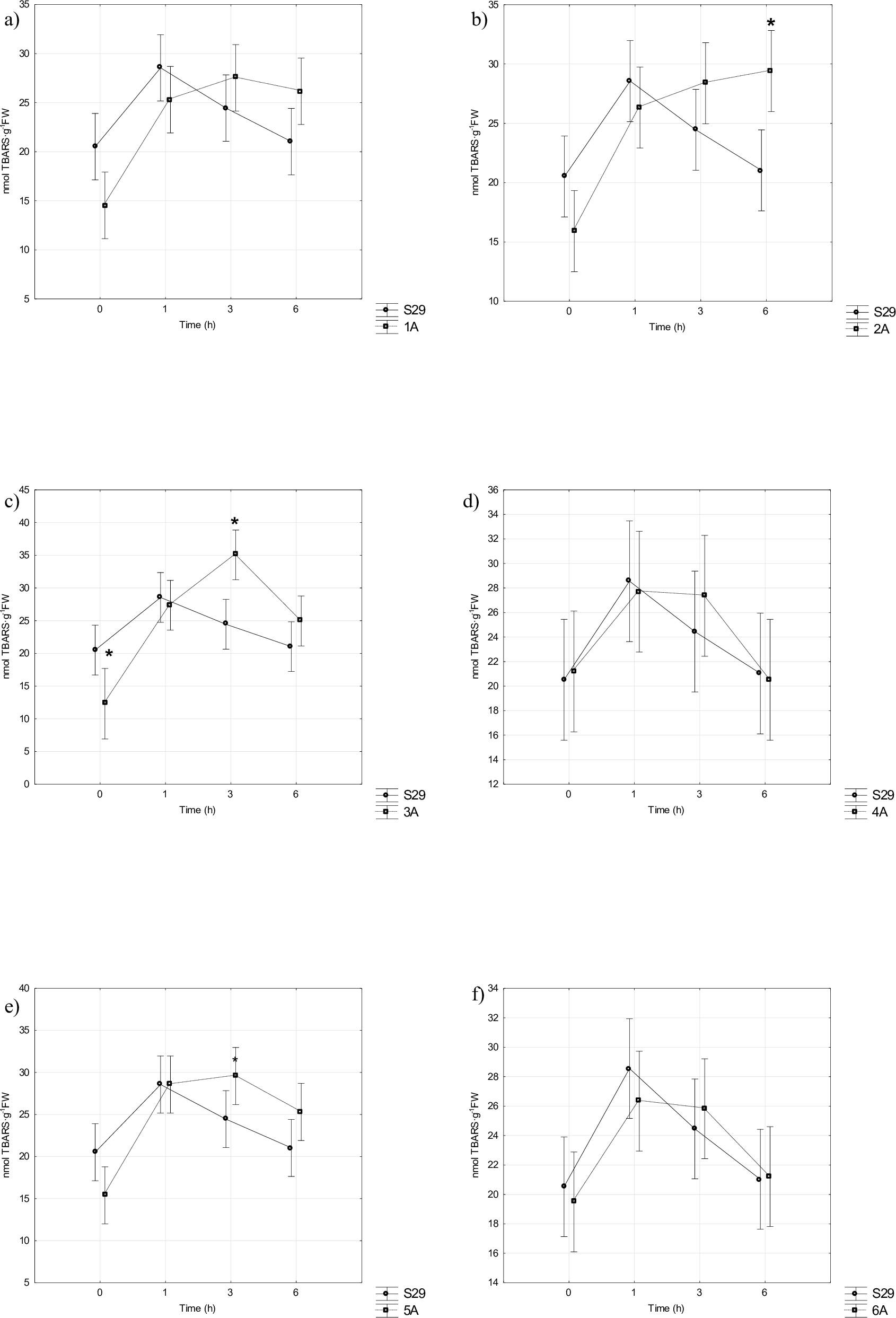

Supplement: S1 Fig — Changes of lipid peroxidation expressed in terms of TBARS concentration in lines with substitution of A genome chromosomes (a-f) compared to S29 during 1, 3 and 6 h of 10% PEG treatment and in non-exposed plants. Bars represent 95% confidence intervals (CI). *indicates significant differences compared to S29 at p<0.05 according to Dunnett's test. (TIF) [file pone.0221849.s001.tif]

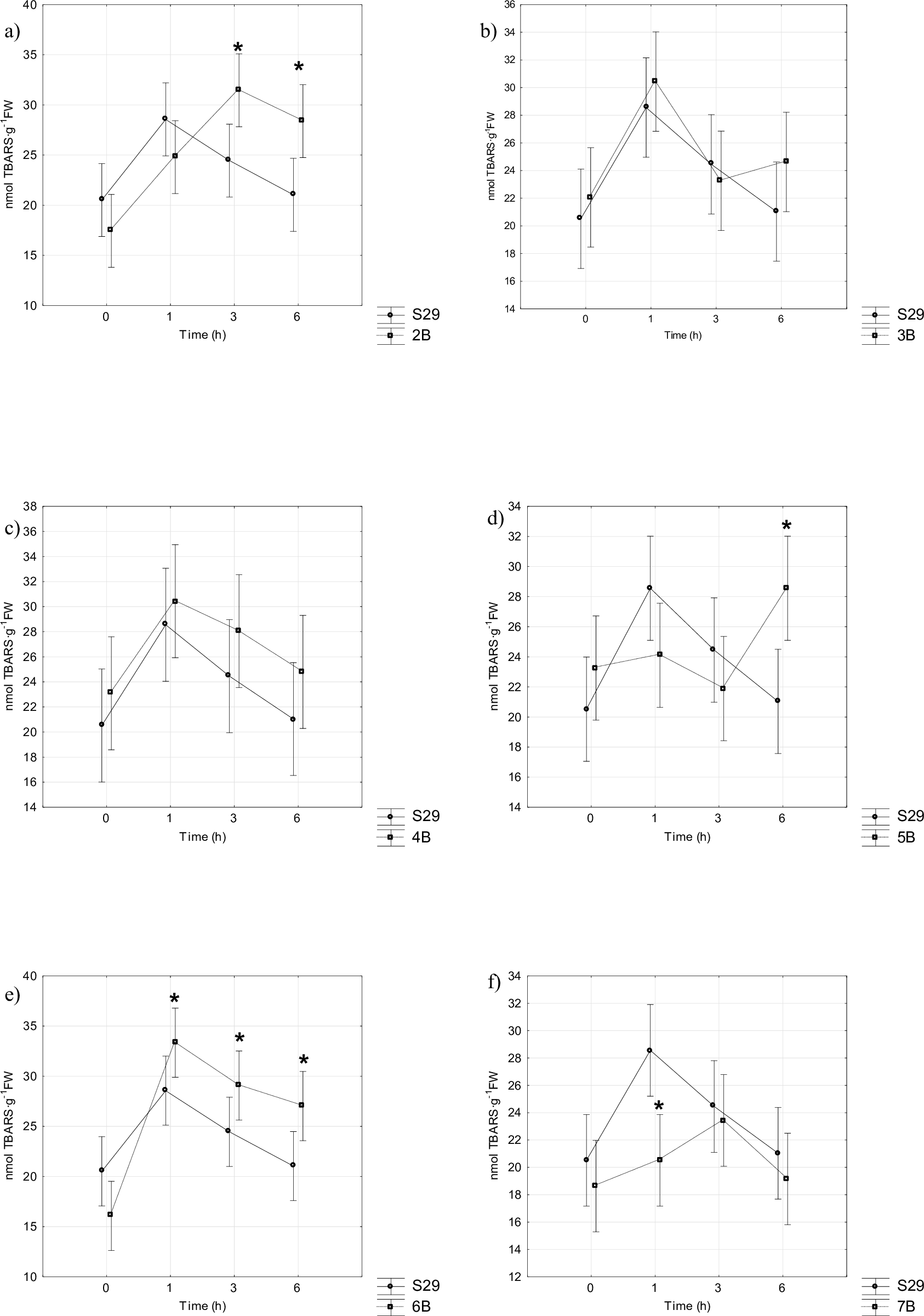

Supplement: S2 Fig — Changes of lipid peroxidation expressed in terms of TBARS concentration in lines with substitution of B genome chromosomes (a-f) compared to S29 during 1, 3 and 6 h of 10% PEG treatment and in non-exposed plants. Bars represent confidence interval (CI). *indicates significant differences compared to S29 at p<0.05 according to Dunnett's test. (TIF) [file pone.0221849.s002.tif]

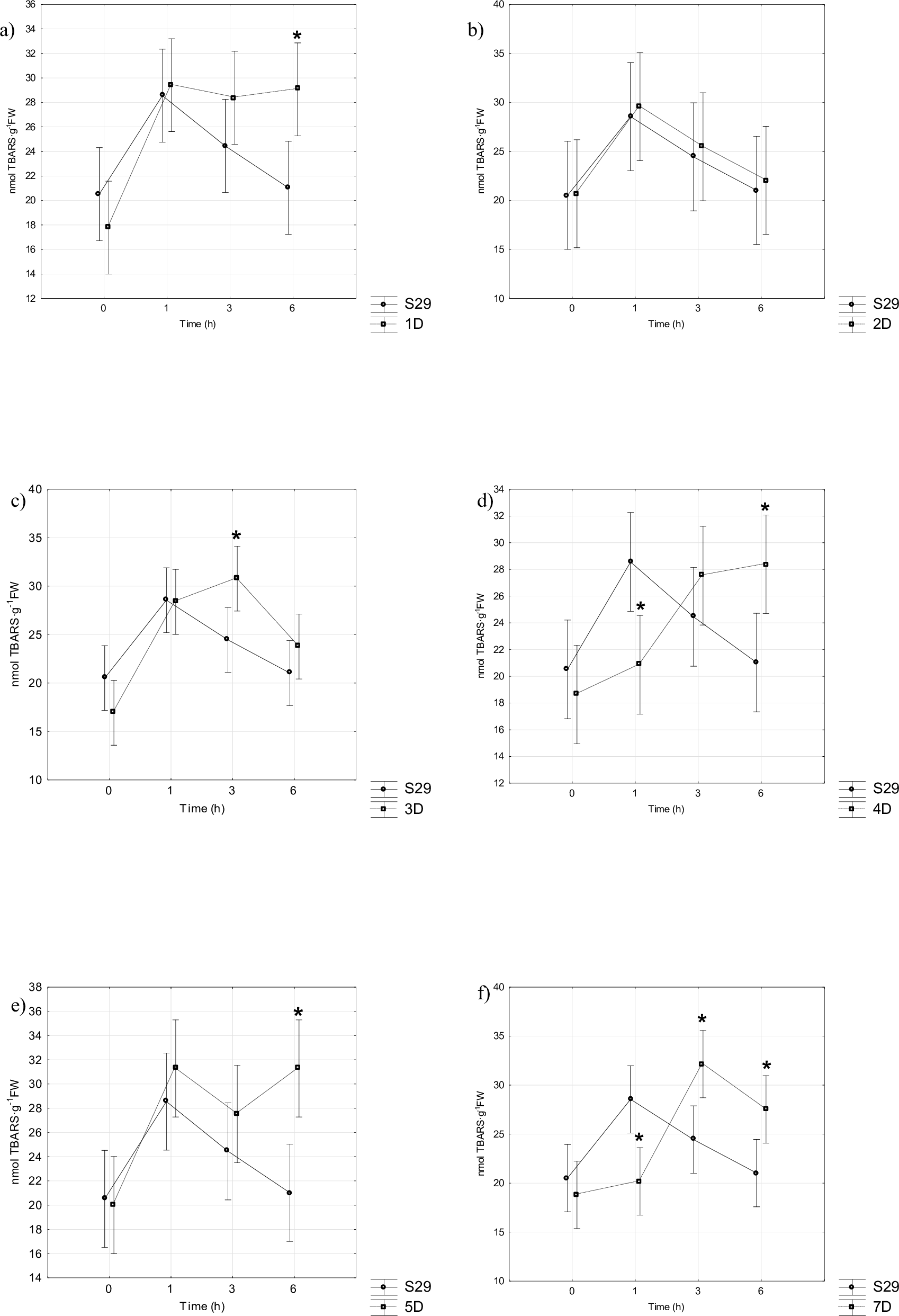

Supplement: S3 Fig — Changes of lipid peroxidation expressed in terms of TBARS concentration in lines with substitution of D genome chromosomes (a-f) compared to S29 during 1, 3 and 6 h of 10% PEG treatment and in non-exposed plants. Bars represent confidence interval (CI). *indicates significant differences compared to S29 at p<0.05 according to Dunnett's test. (TIF) [file pone.0221849.s003.tif]

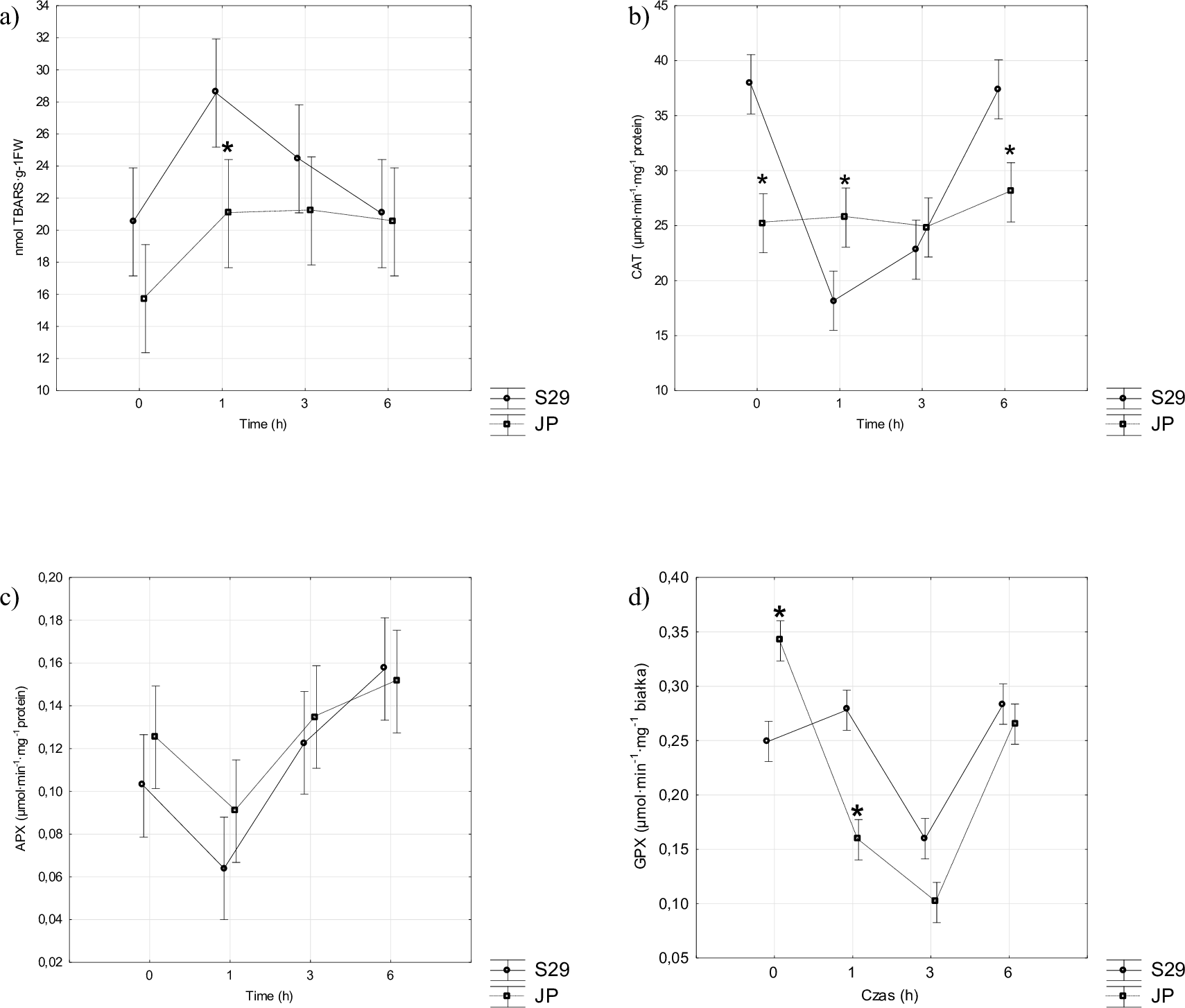

Supplement: S4 Fig — Changes of a) lipid peroxidation expressed in terms of TBARS concentration b) CAT activity c) APX activity d) GPX activity in JP compared to S29 during 1, 3 and 6 h of 10% PEG treatment and in non-exposed plants. Bars represent confidence interval (CI). *indicates significant differences compared to S29 at p<0.05 according to Dunnett's test. (TIF) [file pone.0221849.s004.tif]

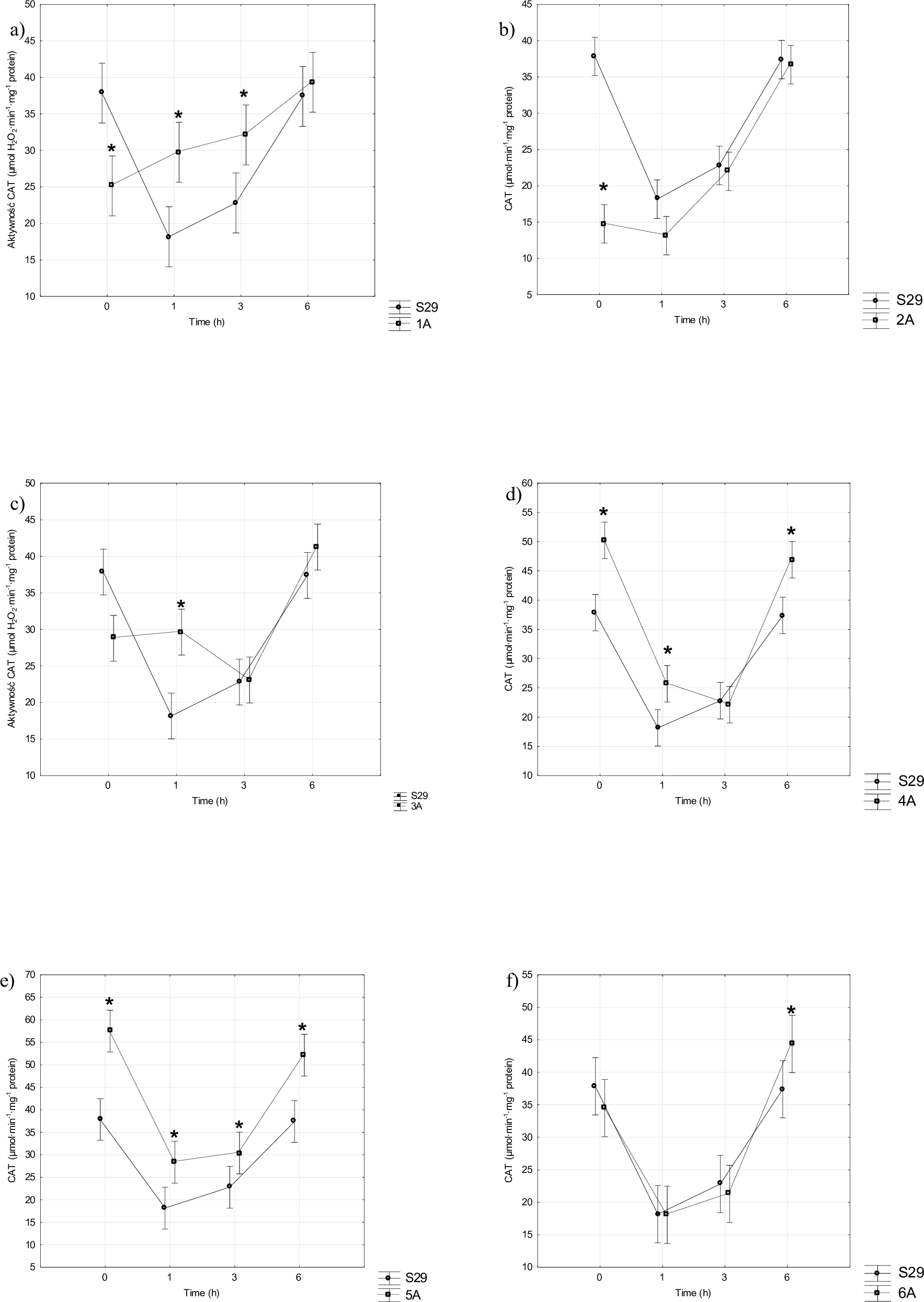

Supplement: S5 Fig — Changes of CAT activity in lines with substitution of A genome chromosomes (a-f) compared to S29 during 1, 3 and 6 h of 10% PEG treatment and in non-exposed plants. Bars represent 95% confidence intervals (CI). *indicates significant differences compared to S29 at p<0.05 according to Dunnett's test. (TIF) [file pone.0221849.s005.tif]

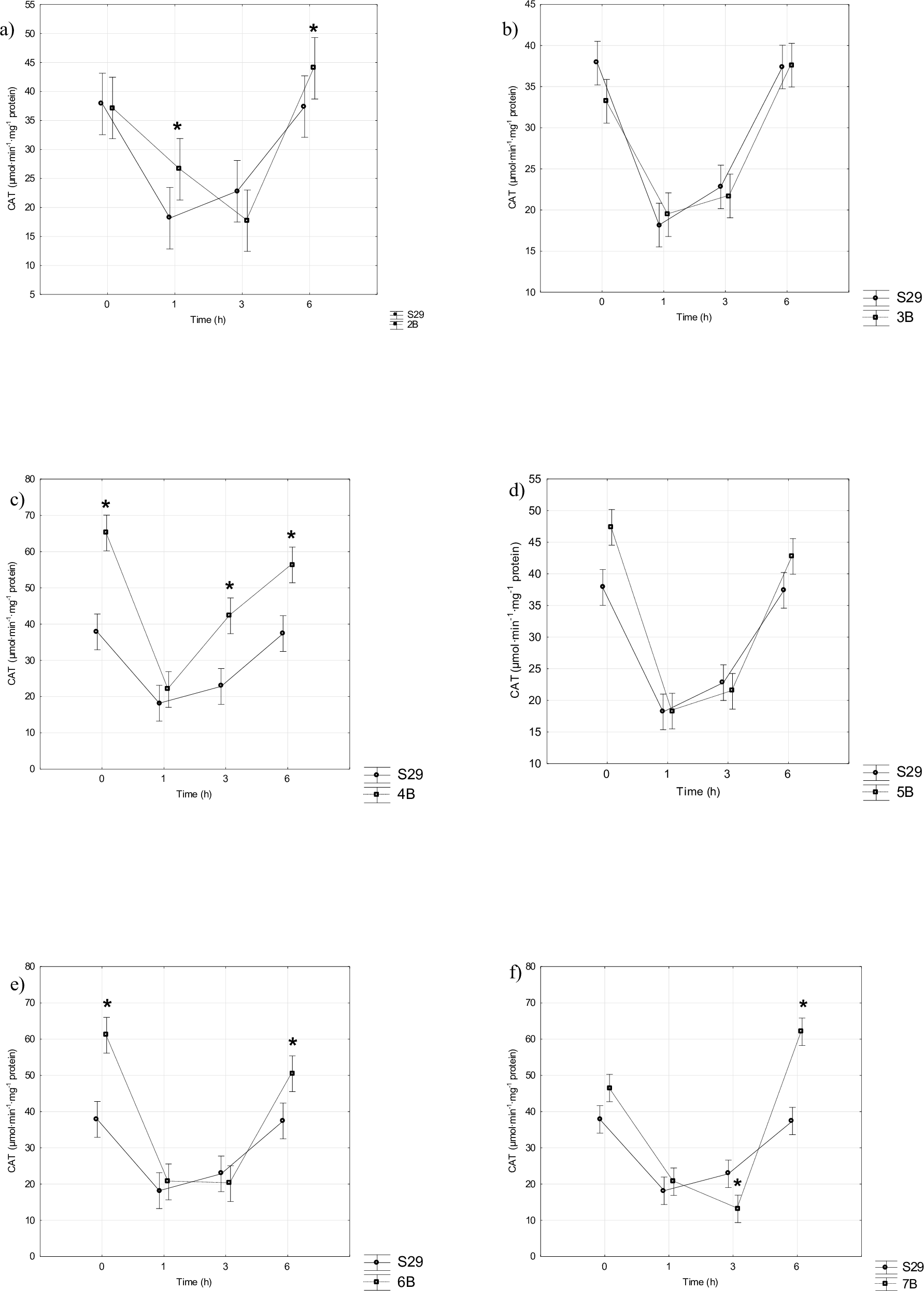

Supplement: S6 Fig — Changes of CAT activity in lines with substitution of B genome chromosomes (a-f) compared to S29 during 1, 3 and 6 h of 10% PEG treatment and in non-exposed plants. Bars represent confidence interval (CI). *indicates significant differences compared to S29 at p<0.05 according to Dunnett's test. (TIF) [file pone.0221849.s006.tif]

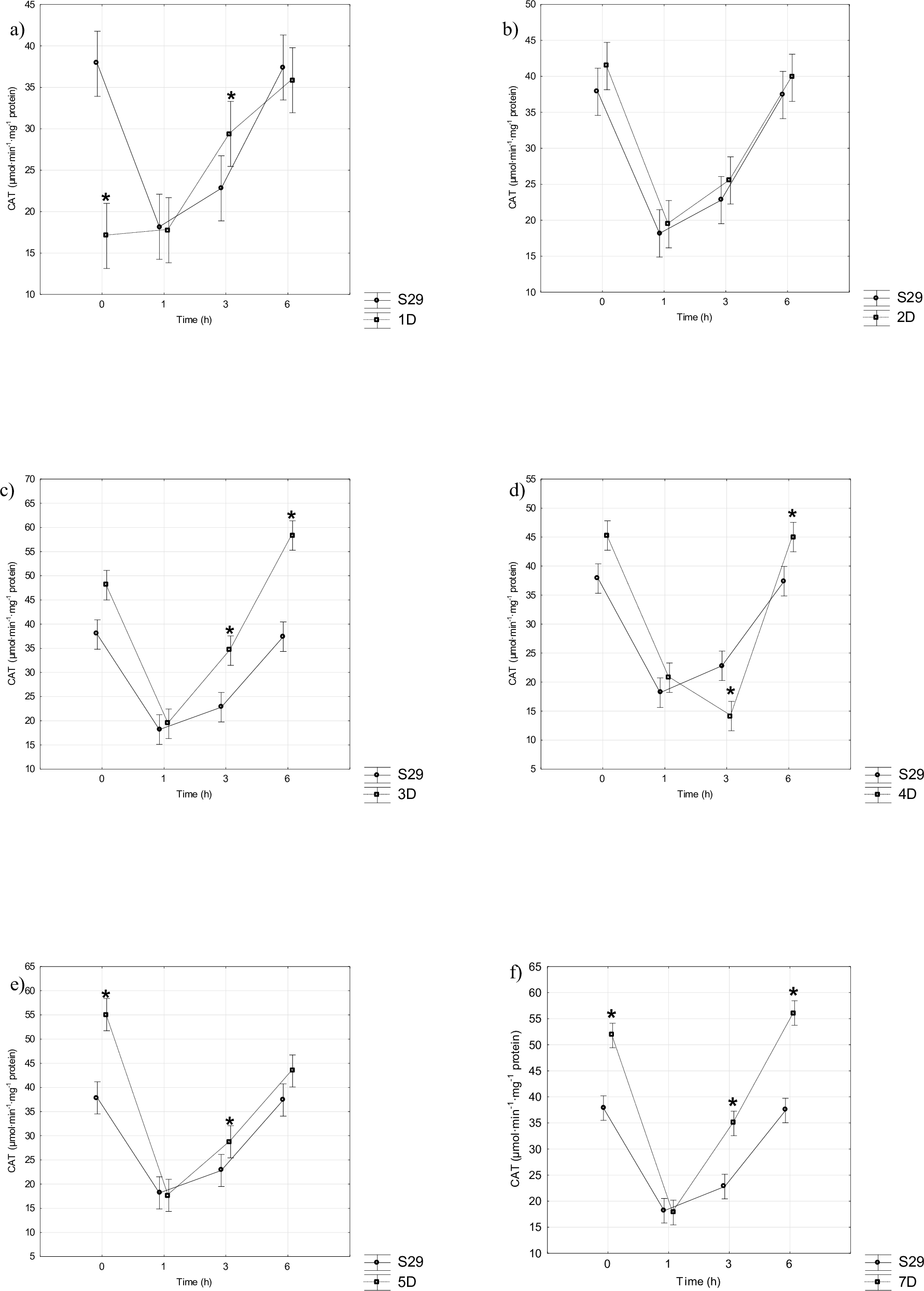

Supplement: S7 Fig — Changes of CAT activity in lines with substitution of D genome chromosomes (a-f) compared to S29 during 1, 3 and 6 h of 10% PEG treatment and in non-exposed plants. Bars represent confidence interval (CI). *indicates significant differences compared to S29 at p<0.05 according to Dunnett's test. (TIF) [file pone.0221849.s007.tif]

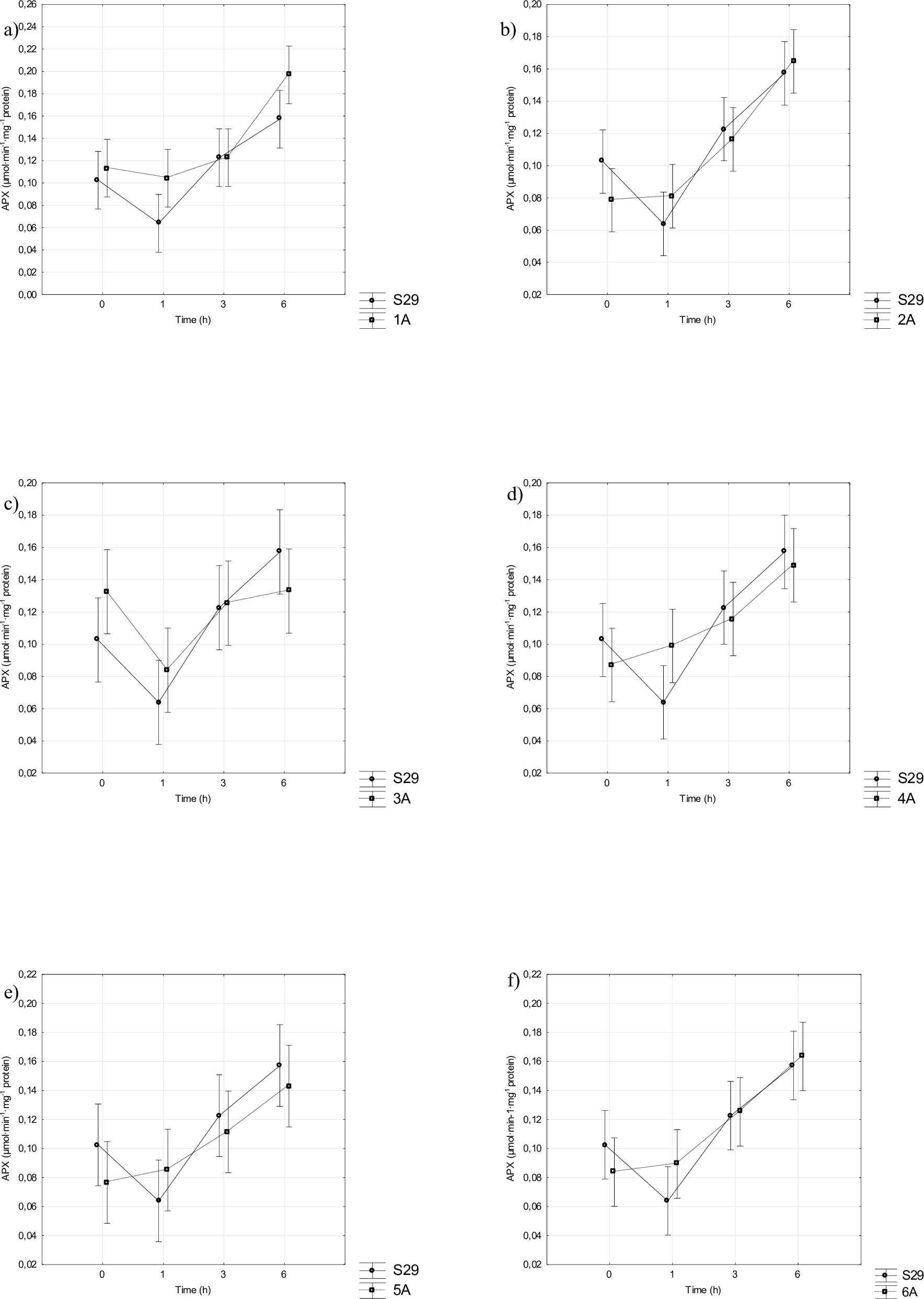

Supplement: S8 Fig — Changes of APX activity in lines with substitution of A genome chromosomes (a-f) compared to S29 during 1, 3 and 6 h of 10% PEG treatment and in non-exposed plants. Bars represent 95% confidence intervals (CI). *indicates significant differences compared to S29 at p<0.05 according to Dunnett's test. (TIF) [file pone.0221849.s008.tif]

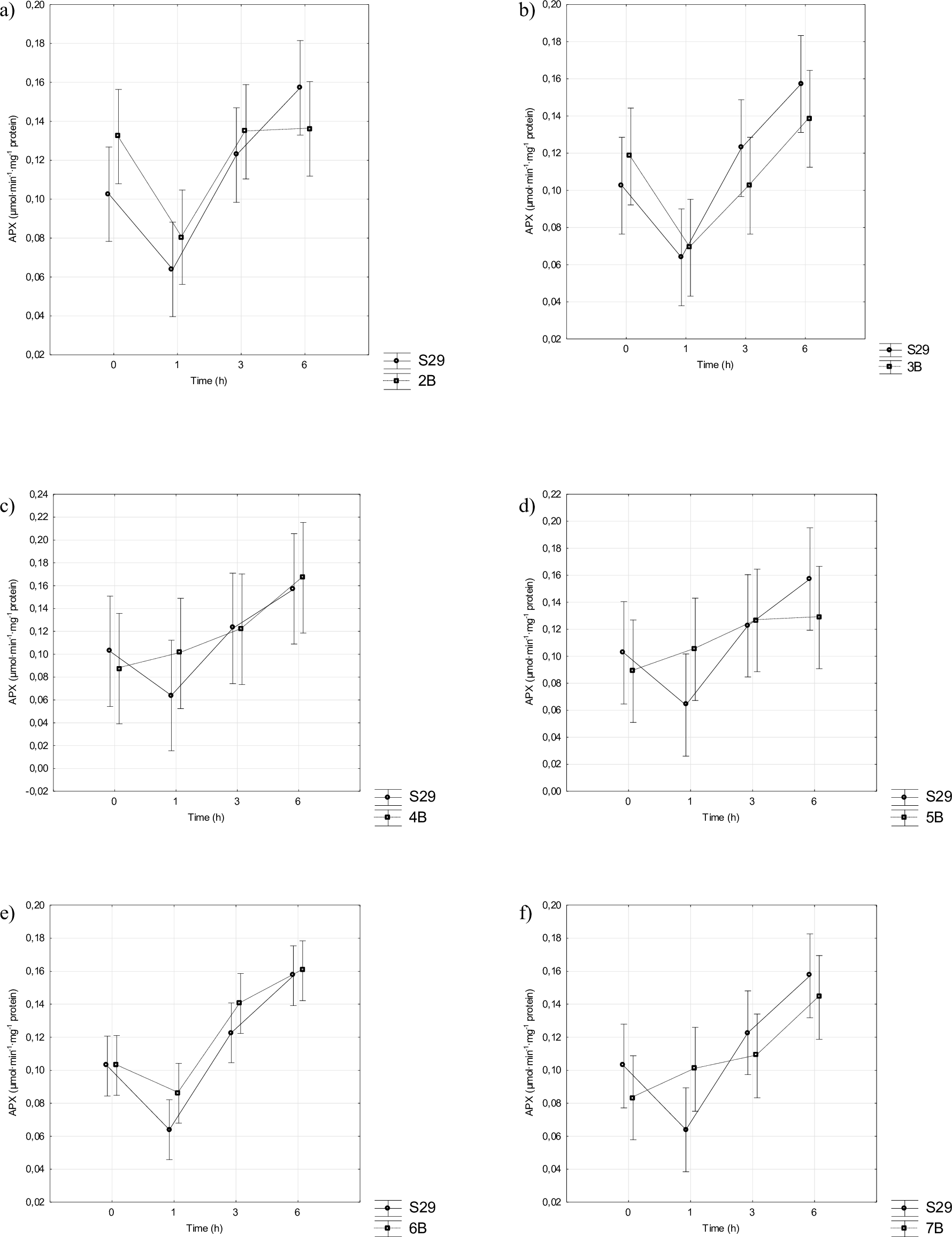

Supplement: S9 Fig — Changes of APX activity in lines with substitution of B genome chromosomes (a-f) compared to S29 during 1, 3 and 6 h of 10% PEG treatment and in non-exposed plants. Bars represent confidence interval (CI). *indicates significant differences compared to S29 at p<0.05 according to Dunnett's test. (TIF) [file pone.0221849.s009.tif]

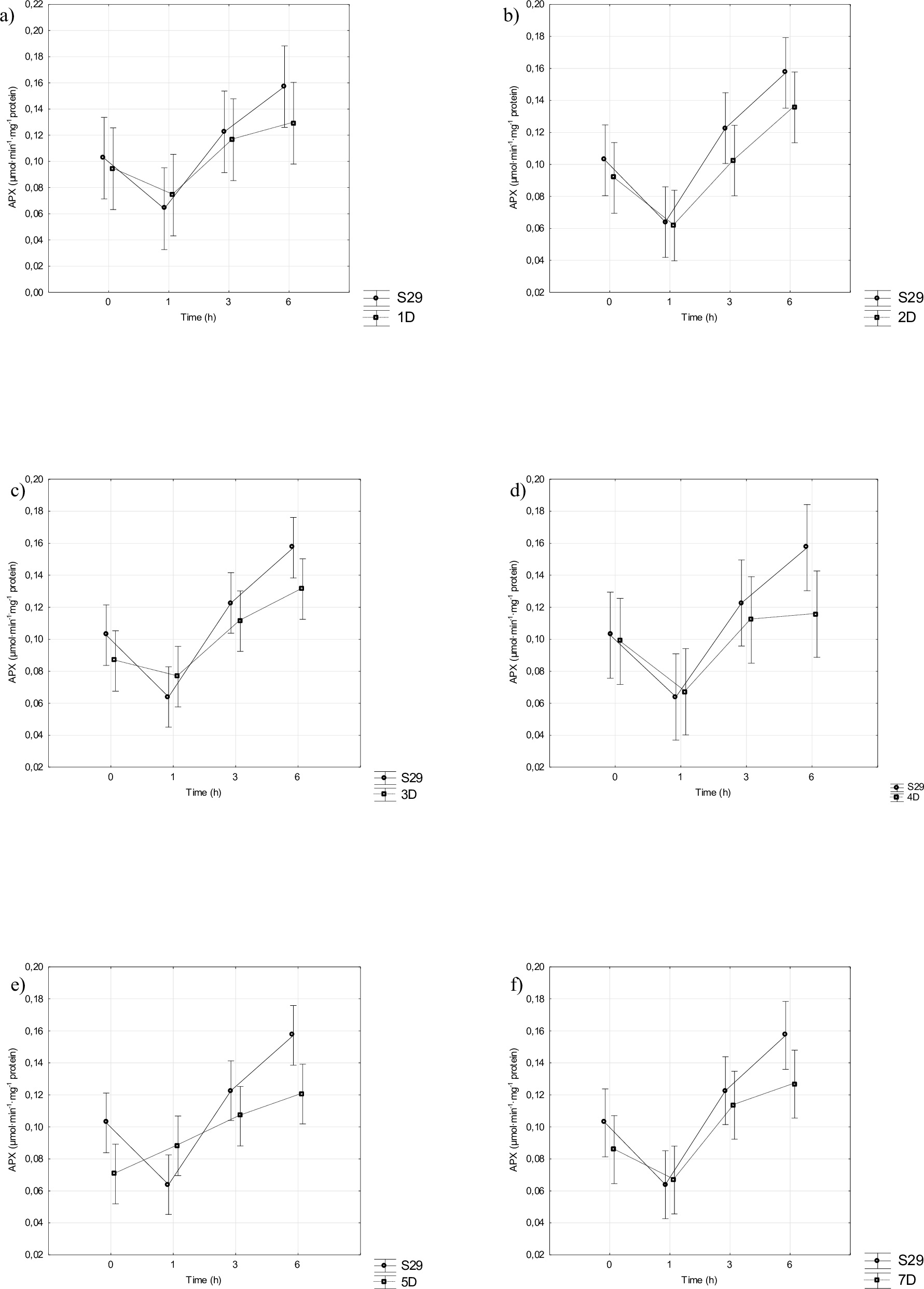

Supplement: S10 Fig — Changes of APX activity in lines with substitution of D genome chromosomes (a-f) compared to S29 during 1, 3 and 6 h of 10% PEG treatment and in non-exposed plants. Bars represent confidence interval (CI). *indicates significant differences compared to S29 at p<0.05 according to Dunnett's test. (TIF) [file pone.0221849.s010.tif]

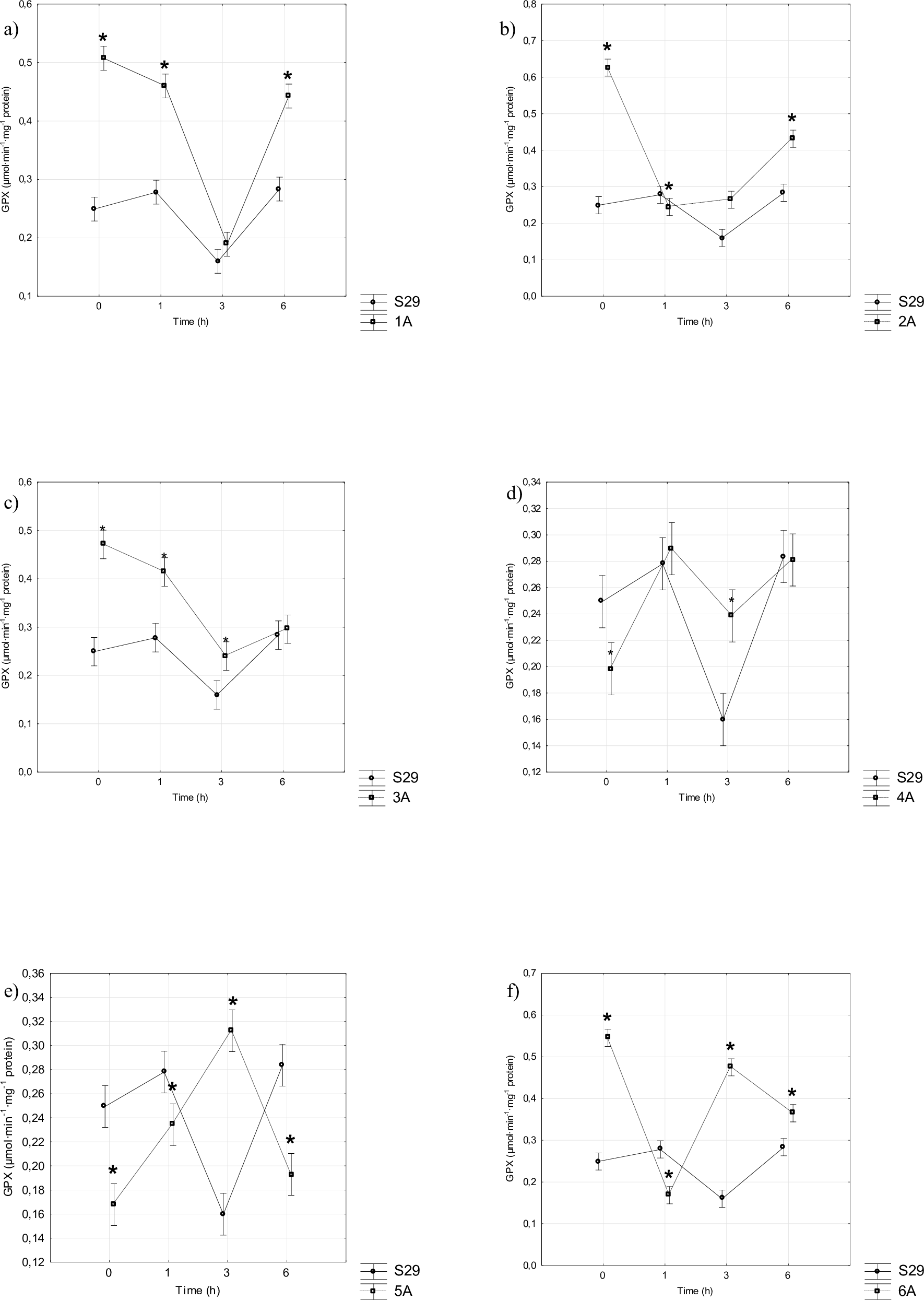

Supplement: S11 Fig — Changes of GPX activity in lines with substitution of A genome chromosomes (a-f) compared to S29 during 1, 3 and 6 h of 10% PEG treatment and in non-exposed plants. Bars represent 95% confidence intervals (CI). *indicates significant differences compared to S29 at p<0.05 according to Dunnett's test. (TIF) [file pone.0221849.s011.tif]

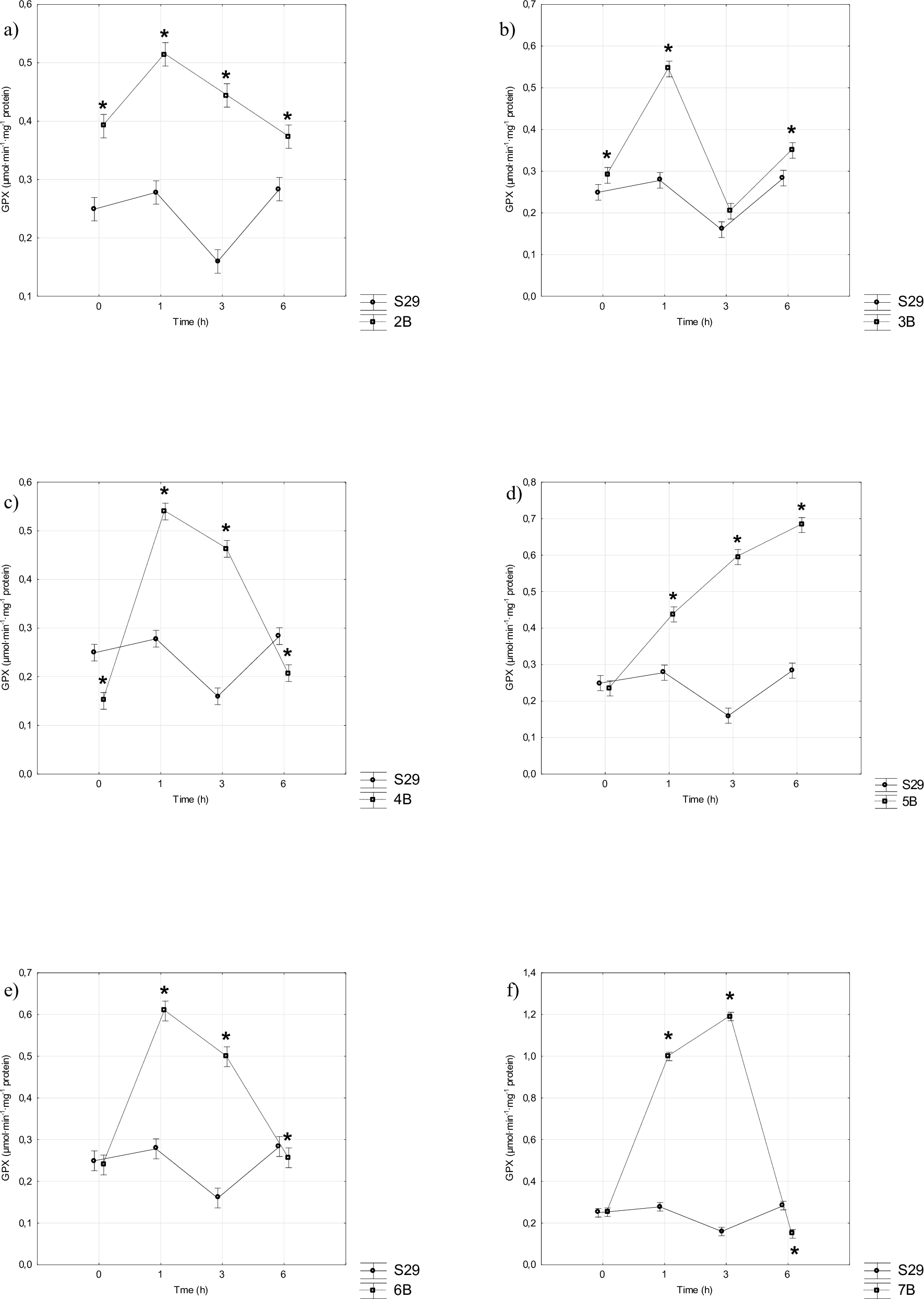

Supplement: S12 Fig — Changes of GPX activity in lines with substitution of B genome chromosomes (a-f) compared to S29 during 1, 3 and 6 h of 10% PEG treatment and in non-exposed plants. Bars represent confidence interval (CI). *indicates significant differences compared to S29 at p<0.05 according to Dunnett's test. (TIF) [file pone.0221849.s012.tif]

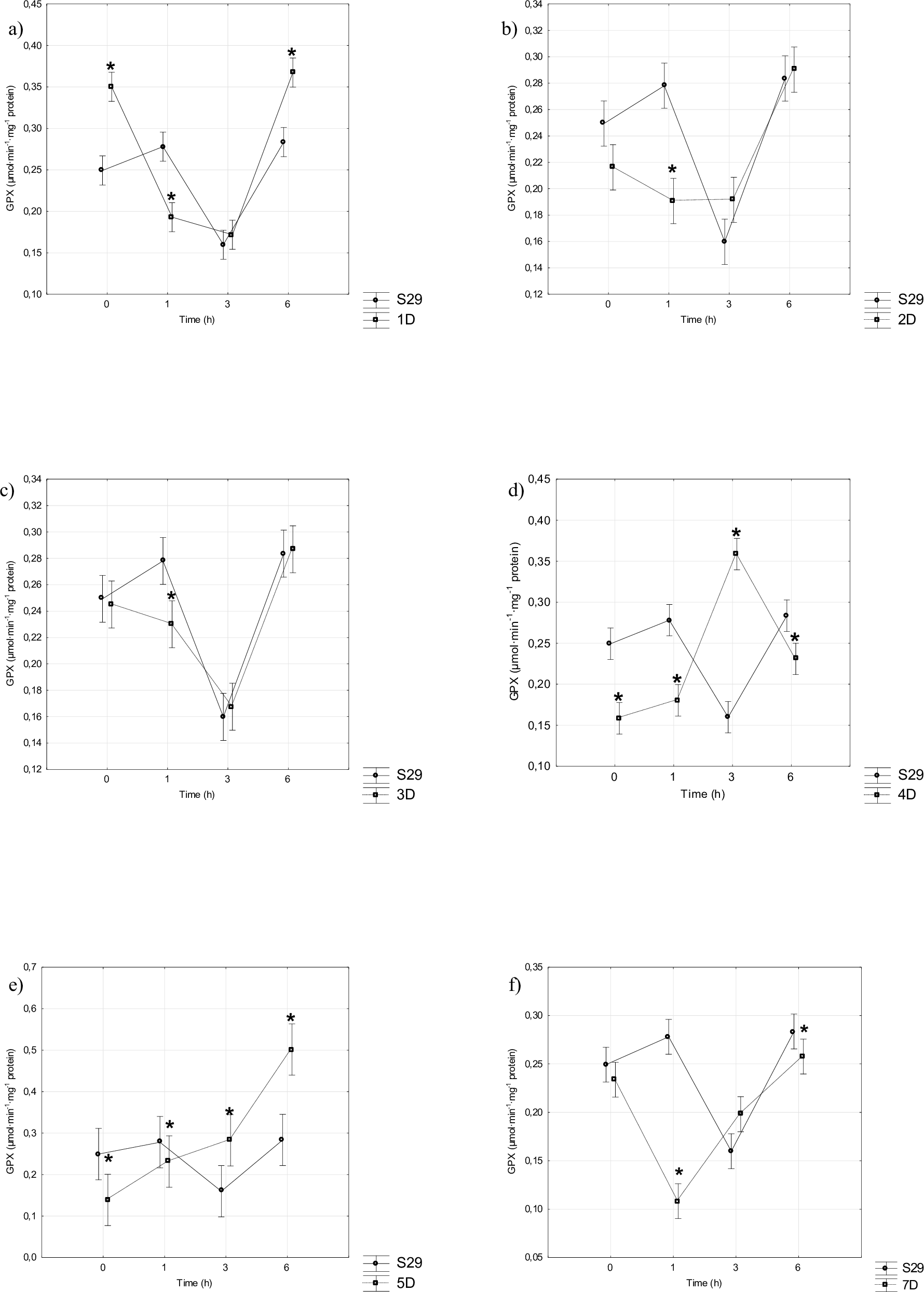

Supplement: S13 Fig — Changes of GPX activity in lines with substitution of D genome chromosomes (a-f) compared to S29 during 1, 3 and 6 h of 10% PEG treatment and in non-exposed plants. Bars represent confidence interval (CI). *indicates significant differences compared to S29 at p<0.05 according to Dunnett's test. (TIF) [file pone.0221849.s013.tif]
