# Supplementary material for: Using intervarietal substitution lines for the identification of wheat chromosomes involved in early responses to water-deficit stress
Source: PLoS One. 2019 Aug 29;14(8):e0221849. doi: 10.1371/journal.pone.0221849 (PMC6715202; doi:10.1371/journal.pone.0221849)
Supplement: S1 Table — (PDF) [file pone.0221849.s014.pdf]

**Line**	**Chromosome substituted from 'Janetzkis Probat'**
S29/JP 1A	1A
S29/JP 2A	2A
S29/JP 3A	3A
S29/JP 4A	4A
S29/JP 5A	5A
S29/JP 6A	6A
S29/JP 2B	2B
S29/JP 3B	3B
S29/JP 4B	4B
S29/JP 5B	5B
S29/JP 6B	6B
S29/JP 7B	7B
S29/JP 1D	1D
S29/JP 2D	2D
S29/JP 3D	3D
S29/JP 4D	4D
S29/JP 5D	5D
S29/JP 7D	7D
